# A Comprehensive Analysis of Replicating Merkel Cell Polyomavirus Genomes Delineates the Viral Transcription Program and Suggests a Role for mcv-miR-M1 in Episomal Persistence

**DOI:** 10.1371/journal.ppat.1004974

**Published:** 2015-07-28

**Authors:** Juliane Marie Theiss, Thomas Günther, Malik Alawi, Friederike Neumann, Uwe Tessmer, Nicole Fischer, Adam Grundhoff

**Affiliations:** 1 Research Group Virus Genomics, Heinrich Pette Institute, Leibniz Institute for Experimental Virology, Hamburg, Germany; 2 Institute for Medical Microbiology, Virology and Hygiene, University Medical Center Hamburg-Eppendorf, Hamburg, Germany; 3 Bioinformatics Service Facility, University Medical Center Hamburg-Eppendorf, Hamburg, Germany; National Cancer Institute, UNITED STATES

## Abstract

Merkel cell polyomavirus (MCPyV) is considered the etiological agent of Merkel cell carcinoma and persists asymptomatically in the majority of its healthy hosts. Largely due to the lack of appropriate model systems, the mechanisms of viral replication and MCPyV persistence remain poorly understood. Using a semi-permissive replication system, we here report a comprehensive analysis of the role of the MCPyV-encoded microRNA (miRNA) mcv-miR-M1 during short and long-term replication of authentic MCPyV episomes. We demonstrate that cells harboring intact episomes express high levels of the viral miRNA, and that expression of mcv-miR-M1 limits DNA replication. Furthermore, we present RACE, RNA-seq and ChIP-seq studies which allow insight in the viral transcription program and mechanisms of miRNA expression. While our data suggest that mcv-miR-M1 can be expressed from canonical late strand transcripts, we also present evidence for the existence of an independent miRNA promoter that is embedded within early strand coding sequences. We also report that MCPyV genomes can establish episomal persistence in a small number of cells for several months, a time period during which viral DNA as well as LT-Ag and viral miRNA expression can be detected via western blotting, FISH, qPCR and southern blot analyses. Strikingly, despite enhanced replication in short term DNA replication assays, a mutant unable to express the viral miRNA was severely limited in its ability to establish long-term persistence. Our data suggest that MCPyV may have evolved strategies to enter a non- or low level vegetative stage of infection which could aid the virus in establishing and maintaining a lifelong persistence.

## Author Summary

MCPyV is a recently discovered human polyomavirus that is likely to cause the majority of cases of Merkel cell carcinoma (MCC), a rare but highly aggressive skin cancer. While much research has been focused on understanding transforming functions of MCPyV gene products, owing to the lack of fully permissive replication systems, the natural lifecycle of the virus is poorly understood. Using high-throughput analyses, here we have interrogated a semi-permissive replication system to study the viral transcription program and elucidate the functions of the viral microRNA (miRNA) mcv-miR-M1. We find that, similar to other polyomavirus miRNAs, mcv-miR-M1 has the ability to negatively regulate expression of viral gene products required for viral DNA replication. Unexpectedly, however, we also observe that mcv-miR-M1 augments long-term episomal persistence of MCPyV genomes. Given that MCPyV establishes persistent infections in the majority of healthy human adults, our observations shed new light on the mechanisms that may be employed by this tumor virus to mount a lifelong chronic infection of its host.

## Introduction

Merkel cell polyomavirus (MCPyV), first identified in 2008 in tissue from Merkel cell carcinoma (MCC) [1], is the only human polyomavirus considered to be the etiological agent of tumors arising in its natural host. Several lines of evidence, including frequent detection of monoclonally integrated sequences bearing hallmark mutations and constitutive expression of T antigens in tumor tissues suggest that the virus is causally linked to MCC pathogenesis (reviewed in [2]). Epidemiological studies suggest that MCPyV infection occurs in childhood and persists for life in the majority of the adult healthy population [3–7]. Hence, the occurrence of MCC is an extremely rare complication of MCPyV infection.

Like all polyomaviruses, MCPyV encodes the early large and small T antigens (LT- and sT-Ag), as well as the late structural antigens VP1 and VP2 [1, 8]. Whether MCPyV also expresses a functional VP3 antigen remains a matter of debate [9]. Polyomavirus T antigens are produced via alternative splicing from a single gene cassette that is transcribed early during infection. In MCPyV, alternative splicing of early transcripts additionally produces a 57K T antigen of hitherto unknown function [8]. A recent study furthermore revealed the existence of an alternative open reading frame (ALTO) which can be produced by leaky scanning of T-Ag encoding transcripts [10]. Although ALTO shares certain sequence features with the middle T antigens (mT-Ag) of other polyomaviruses, its precise functions remain unknown. Experimental evidence suggests that presence or absence of ALTO does not affect viral DNA replication [10].

In addition to above protein products, MCPyV has been found to encode a single microRNA (miRNA) precursor which can produce two mature miRNAs, termed mcv-miR-M1-5p and -3p [11]. miRNAs are small (~22 nt.), non-coding RNAs that can be produced from primary transcripts via sequential processing by the nucleases Dicer and Drosha [12]. After incorporation into the RNA-induced silencing complex (RISC), mature miRNA can negatively regulate the expression of transcripts that are recognized via sequence complementarity. In animals, target site recognition is primarily guided by perfect Watson-Crick pairing of the so-called seed sequence (nucleotides 2–8) of the miRNA, whereas the distal sequences typically only exhibit poor sequence complementarity [13]. This partial pairing leads to translational inhibition of the mRNA (although frequently a modest reduction in overall transcript levels can also be observed). Although rarely seen for animal miRNAs, plant miRNAs as well as miRNAs encoded by some animal viruses can also bind to their targets with perfect complementarity, resulting in RISC-mediated endonucleolytic cleavage of the mRNA.

Recent studies have shown that a number of human and animal polyomaviruses encode miRNAs [11, 14–19]. Although their precise genomic location varies, all known polyomavirus miRNA are expressed from sequences that are located in antisense orientation to the early T antigen encoding transcripts. Consequently, mature miRNA species expressed from these loci exhibit perfect complementarity to early transcripts, and a number of studies suggest that all hitherto identified polyomavirus miRNAs share the ability to negatively regulate expression of early gene products [11, 15–17, 19, 20]. For most of the known PyV miRNAs (including MCPyV), experimental evidence for the above is limited to ectopic heterologous reporter systems. However, miRNA-knockout viruses have been generated for SV40, murine PyV and BKPyV, and *in vitro* studies using such viruses demonstrated that the viral miRNAs are indeed able to efficiently limit LT-Ag expression as well as DNA replication in the context of authentic episomes [15, 16, 20]. So far, experimental *in vivo* infections with miRNA-deficient viruses have only been performed for SV40 and murine PyV [15, 21]. Indeed, miRNA-deficient SV40 mutants produce consistently higher viral DNA loads in both liver and kidney of infected syrian golden hamsters when compared to wt viruses. However, both wt and mutant viruses were able to establish persistent infections, and thus far only limited evidence for increased clearance of miRNA-mutants has been observed [21]. In the case of murine PyV, the kinetics of both infection establishment as well as subsequent viral clearance in experimentally inoculated mice were comparable between wt and mutant viruses, indicating that (at least under the experimental conditions used) murine PyV miRNA expression is not essential for the infection of mice [15]. The above therefore suggests that the role of PyV miRNAs during natural infection may involve aspects of acquisition, spread or persistence which are not properly recapitulated by the experimental *in vivo* systems used. Hence, while evolutionary conservation suggests important function for miRNA-mediated autoregulation of LT-Ag expression and DNA replication, the precise selectional advantage conferred by this regulatory mechanism remains unclear [22–25].

The molecular mechanisms that lead to polyomavirus miRNA expression thus far have not been studied in much detail. Circumstantial evidence, however, suggests that at least in some polyomaviruses transcriptional read-through beyond weak late strand polyadenylation signals can generate primary RNA molecules that traverse the miRNA precursor sequences [15–17]. In such a model, miRNA expression is coupled to expression of coding transcripts that originate from the late promoter in the non-coding control region (NCCR). Indeed, a recent study of BK polyomavirus (BKPyV) has demonstrated that NCCR rearrangements which naturally arise in patients suffering from BKPyV-associated disease result in decreased late strand transcription and miRNA expression [20]. In contrast, archetype viruses express robust levels of the viral miRNA, which in turn dampens T antigen expression and viral replication. As the archetype virus is thought to be responsible for establishment of persistent urinary tract infections, these findings suggest that, similar to herpesviruses, polyomaviruses may employ miRNAs to facilitate chronic infection of their host [20, 26]. Whether similar mechanisms as the above may dictate viral miRNA expression in MCPyV, a virus that is only distantly related to BKPyV, has thus far not been elucidated.

Given its association with human tumors, experimental research on MCPyV thus far has been largely focused on growth promoting and transforming functions of early viral gene products. In contrast, there is a profound lack of knowledge regarding the natural life cycle of the virus. In large part, this is due to the fact that all currently available *in vitro* systems produce only very low titers of viral progeny [27–30]. Although recent evidence suggests that MCPyV may persist in the hematopoietic compartment [31–33], it is unknown which type of cell may support viral replication and/or serve as a reservoir for persistent infection *in vivo*. It is therefore also unclear whether the low transmissibility observed *in vitro* reflects an inherent property of the virus (e.g., similar to what is observed for archetype BKPyV) or simply results from the lack of appropriate cell culture systems.

In addition to (and partially as a result of) the above deficits, there is only very limited knowledge regarding the MCPyV transcription program. Thus far, experimental studies addressing this subject have mainly employed subgenomic MCPyV fragments under the control of heterologous promoters to study expression and processing of the viral miRNA, or to explore the structure and coding potential of early region transcripts [8, 11]. Additionally, endogenous expression of early gene products and the viral miRNA has been investigated in MCC-derived cell lines (MCCL) or MCC tissues [11, 19, 34, 35]. These studies have shown that the defective viral genomes integrated in MCC constitutively express proteins encoded by the early region, but only produce the viral miRNA at low levels. Thus, it remains unknown whether intact episomal MCPyV genomes express the miRNA at levels which permit efficient autoregulation of LT-Ag expression and viral DNA replication.

We have previously established a semi-permissive replication system which is based on synthetic MCPyV genomes (MCVSyn) that are 100% identical to prototypical field strain sequences [27]. After transfection, viral genomes undergo active DNA replication, express early and late antigens, and produce infectious progeny (albeit at very low titers). Here, we have used the above system to study the viral transcription program and elucidate expression mechanisms and functions of the viral miRNA during short and long term culture of cells harboring actively replicating episomes.

## Results

### Small RNA-seq demonstrates high-level expression of mcv-miR-M1 by replicating MCPyV episomes and argues against the existence of tumor-specific miRNA moieties

**Fig 1:**
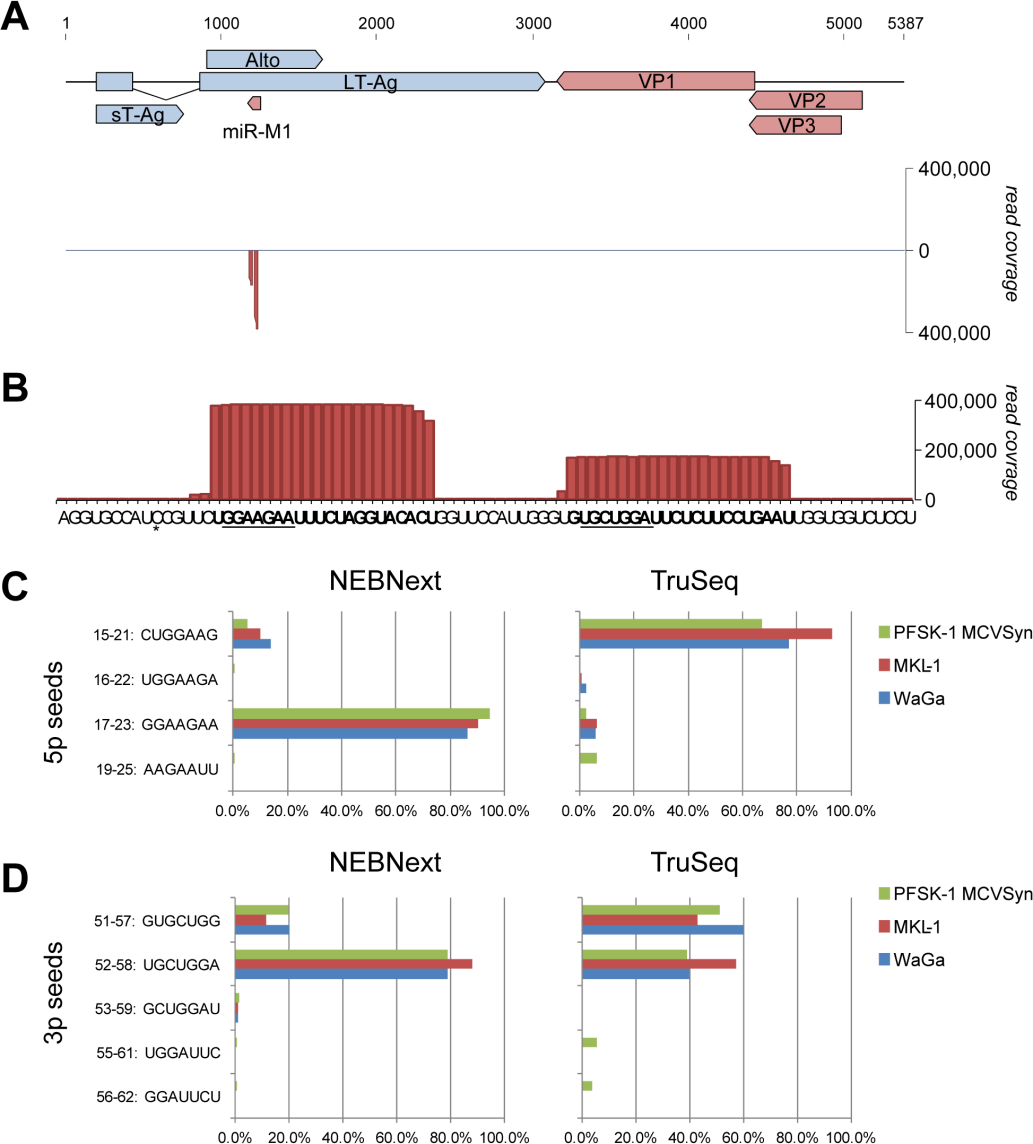
Expression of mcv-miR-M1 by replicating MCPyV genomes. **(A)** *Top*: Schematic illustration of the MCPyV genome. Early (light blue) and late (red) strand open reading frames as well as the mcv-miR-M1 locus are shown as solid block arrows. The 57K T-Ag ORF is not shown for simplicity. *Bottom*: Small RNA read coverage on early (positive axis) or late (negative axis) strands as observed in MCVSyn transfected PFSK-1 cells (NEBNext library preparation protocol). **(B)** Enlarged depiction of late strand coverage at the genomic mcv-miR-M1 locus as shown in (A). The regions producing the major 5p and 3p mature miRNAs are shown in bold and their seed sequences are underlined. The asterisk denotes a single nucleotide polymorphism that is frequently observed in MCPyV field strains. **(C, D)** Relative frequency of mature 5p (C) or 3p (D) mcv-miR-M1 reads containing the indicated seed sequences in small RNA libraries prepared with the NEBNext (left panels) or TruSeq (right panels) library preparation protocols.

Ectopic expression of a select number of computationally predicted pre-miRNA candidates previously identified a single pre-miRNA hairpin (termed mcv-miR-M1) encoded by MCPyV [11], located in an antisense orientation to the early coding region at genomic coordinates 1168 to 1251 (Fig 1A). While low-level expression of mature miRNAs from this genomic locus has been confirmed in primary MCC tissues [34], an unbiased investigation of small RNAs produced from intact and replicating viral episomes had previously not been performed. We therefore sought to determine i) whether the previously identified miRNA mcv-miR-M1 is expressed at significant levels by replicating episomes, ii) whether mcv-miR-M1 is the only miRNA expressed by authentic MCPyV genomes and iii) whether mature mcv-miR-M1 moieties may undergo differential processing in MCC-derived cell lines (MCCL). The latter point was of particular interest given that a recent study reported MCC-derived mature 5p miRNAs that differed from those described by Seo et al. in a 2 nt. shift, resulting in an altered seed sequence and a therefore a differential set of predicted cellular target transcripts [11, 34].

To investigate above questions, we transfected the neuroectodermal tumor cell line PFSK-1 cells with MCVSyn, a viral genome that is 100% identical to prototypical field strain sequences [27]. Small RNA moieties were harvested after 4 days of transfection and subjected to high throughput sequencing using the NEBNext library preparation protocol. As shown in Fig 1A, the great majority (99.5%) of all MCVSyn-derived small RNAs mapped to the previously identified mcv-miR-M1 locus. The remaining reads were randomly scattered across the viral genome (not visible at the scale shown in Fig 1A; see S1 Dataset for complete coverage data), suggesting they represent random mRNA breakdown products. Hence, the previously identified mcv-miR-M1 is the only miRNA expressed by actively replicating MCPyV genomes.

Comparison of viral and human miRNA read counts suggests that mcv-miR-M1-derived miRNAs are highly expressed in MCVSyn-transfected PFSK-1 cells, accounting for approximately 3% of the total of 19.3 million mature miRNA reads (Table 1). Mature miRNAs derived from the 5'-arm of the pre-miRNA hairpin were approximately twofold more abundant than those derived from the 3'-arm (mcv-miRs-M1-5p and -3p, respectively, in Table 1). Even though transfection efficiencies achieved with re-circularized genomes were generally below 5%, mcv-miRs-M1-5p and -3p were the 7th and 14th most highly expressed miRNAs, respectively, amongst all mature miRNAs detected in PFSK-1:MCVSyn cultures (Table 2 and S2 Dataset).

**Table 1:** Small RNA-seq summary statistics.

	PFSK-1:MCVSyn		WaGa		MKL-1	
miRNA reads	NEBNext	TruSeq	NEBNext	TruSeq	NEBNext	TruSeq
total^**a**^	19,290,137	34,156,074	13,394,824	59,645,518	17,427,420	54,938,374
human^**b**^	18,725,363	34,117,198	13,394,674	59,645,419	17,427,233	54,938,222
mcv-miR-M1	564,774	38,876	150	99	187	152
-5p species	388,830	30,553	59	82	87	129
-3p species	175,944	8,323	91	17	100	23
-% of total reads	2.93%	0.11%	0.0011%	0.0002%	0.0011%	0.0003%

***a***: total number of reads mapped to human or MCPyV miRNAs

***b***: number of reads mapped to human miRNAs in release 21 of the miRBase registry

**Table 2:** mcv-miR-M1-5p/3p ranks.

	PFSK1:MCVSyn		WaGa		MKL-1	
miRNA species	NEBNext	TruSeq	NEBNext	TruSeq	NEBNext	TruSeq
mcv-miR-M1-5p	7	101	467	529	442	399
mcv-miR-M1-3p	14	159	414	839	416	695

Numbers indicate ranks of mcv-miR-M1-5p and -3p relative to human miRNAs according to expression values.

As shown in Fig 1B, in perfect accord with the original findings by Seo et al. we find that the majority of mcv-miR-M1-5p reads are derived from nucleotides 16–37 of the pre-miRNA hairpin. The seed sequence (GGAAGAA) in this miRNA extends from nucleotide 17–23 of the pre-miRNA (underlined in Fig 1B). Overall, mature miRNAs with this seed (referred to as 5p_17-23_ species in the following) accounted for greater than 94% of all 5p reads (left panel in Fig 1C, green bars, and S3 Dataset). We additionally detected alternatively processed mature 5p species (isomiRs) of lower abundance. Although the mature miRNA species identified by Lee and colleagues (seed sequence CUGGAAG, termed 5p_15-21_ in the following) in MCC tissues was the most prominent among these, its relative abundance accounted for only 5.5% of all 5p reads. To investigate whether these miRNA species may be more abundant in MCC-derived cells, we performed additional small RNA sequencing from the MCPyV-positive MCCLs WaGa and MKL-1. In both cell lines, the frequency of mcv-miR-M1-derived miRNAs was more than 3 orders of magnitude lower than in MCVSyn-transfected PFSK-1 cells (approx. 0.001% of all mature miRNA reads, see Table 1). However, the relative distribution of seed sequences was very similar to that seen in PFSK-1 MCVSyn cells, with the seed sequence observed by Lee being only marginally abundant at 10 to 14% (red and blue bars in the left panel of Fig 1C).

We hypothesized that a potential bias during library preparation might have been responsible for the discrepancies between our or Seo et al.'s results and those observed by Lee and colleagues. It is well documented that biases especially during the ligation step can result in a gross underrepresentation of individual miRNA species between different library preparation methods [36–40]. To formally investigate this possibility, we re-sequenced the same small RNA material using the standard Illumina TruSeq small RNA library preparation kit. Indeed, as shown in the right panel of Fig 1C this analysis primarily recovered mature miRNAs of type 5p_15-21_. Importantly, however, as in the first set of experiments, the observed seed distribution was similar between PFSK-1 MCVSyn, WaGa and MKL-1 cells, demonstrating that MCPyV miRNAs do not undergo differential processing in MCC-derived cell lines. Generally, normalized read counts for 5p_15-21_ miRNAs were comparable between the two library preparation methods while those for 5p_17-23_ species were about 100fold less abundant in the TruSeq experiments (S3 Dataset), suggesting that the observed differences in relative seed distributions were likely due to failure to retrieve 5p_17-23_ species during TruSeq library preparation.

As for mature 5p miRNAs, 3p miRNA species also showed a differential seed sequence distribution depending on whether libraries were prepared with NEBnext or TruSeq protocols (Fig 1D). Again, however, we observed no major differences between miRNA processing in MCCL or PFSK-1 MCVSyn cells. The most abundant 3p species in the NEB dataset mapped to nts. 51–72 of the mvc-mir-M1 hairpin (seed sequence 52–58: UGCUGGA, see Fig 1B and 1D), whereas in the TruSeq data, reads were more evenly distributed between this miRNA species and an isomiR offset by -1 nucleotide.

### mcv-miR-M1 negatively regulates LT-Ag expression and limits DNA replication of authentic viral episomes

**Fig 2:**
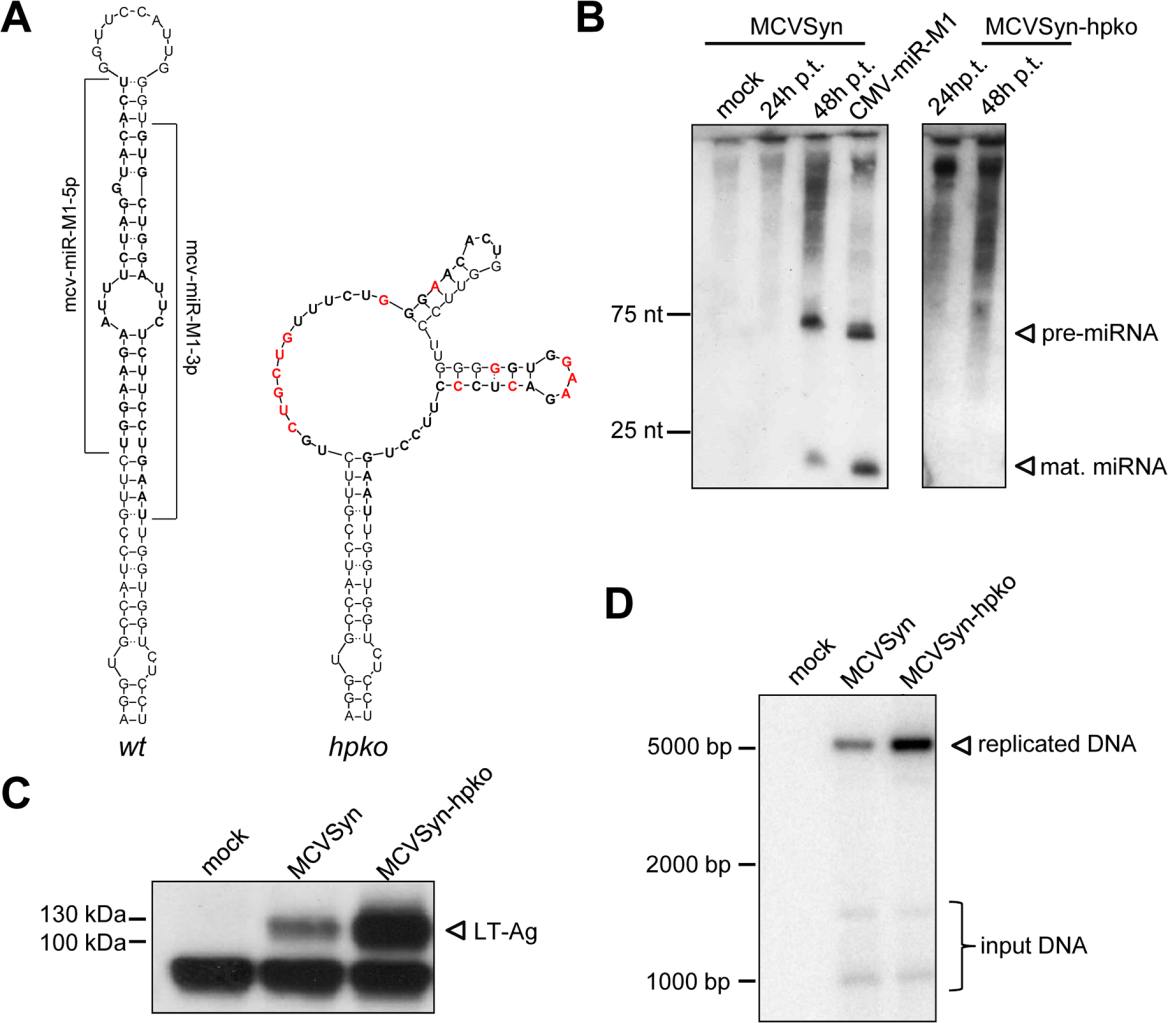
A mcv-miR-M1 knockout mutant exhibits increased LT-Ag expression and enhanced viral DNA replication. **(A)** Predicted secondary structures of the wt (left) and mutated (right) mcv-miR-M1 pre-miRNA sequences. Regions encoding mature miRNAs are shown in bold, mutated nucleotides are highlighted in red. **(B)** Small RNA Northern Blot analysis of mcv-miR-M1 expression 24 and 48 hours after transfection of MCVSyn (left panel) or MCVSyn-hpko (right panel) genomes. Mock transfected cells or cells transfected with a plasmid containing the mcv-miR-M1 pre-miRNA cloned behind a CMV promoter served as negative and positive controls, respectively. Positions of pre- and mature miRNAs are marked by arrows. **(C)** Western Blot analysis of LT antigen expression in PFSK-1 cells transfected with MCVSyn or MCVSyn-hpko at 4d post transfection. The position of LT-Ag is marked by an arrow. **(D)** Analysis of *de novo* replicated viral DNA by Southern Blot analysis of HIRT extracts prepared from PFSK-1 cells transfected with MCVSyn or MCVSyn-hpko genomes 4d post transfection. The position of *DpnI*-resistant replicated DNA is marked with an arrow. Faster migrating bands representing digestion products of the *DpnI*-sensitive input DNA are visible near the bottom of the blot.

Regardless of their exact seed sequence, all mature miRNA species are perfectly complementary to transcripts originating from the opposite strand of the MCPyV genome. Consequently, provided they are efficiently incorporated into RISC, all species should be able to negatively regulate early transcripts. Indeed, a number of previous studies have suggested that the ability to autoregulate LT-Ag expression may represent an evolutionary conserved function of polyomavirus miRNAs [11, 15–17, 19, 20, 22–25]. In support of this notion, Seo and colleagues have formally demonstrated that ectopically expressed mcv-miR-M1 can negatively regulate luciferase expression of a chimeric reporter construct containing the mcv-mir-M1 complementary region and flanking sequences [11]. To determine whether mcv-mir-M1 also can suppress LT-Ag expression in the context of transcription from intact episomes, we generated a mutant MCPyV genome unable to express the viral miRNA. To this end, we introduced a total of 14 mutations designed to disrupt the mcv-mir-M1 pre-miRNA hairpin structure (Fig 2A) which is required for the processing of pre- and mature miRNAs by Drosha and Dicer, respectively. All nucleotide substitutions were designed such that the coding capacity of LT-Ag encoded on the opposite strand remained unaltered (S1 Fig). The resulting viral genome (referred to as MCVSyn-hpko in the following) or the parental MCVSyn genome was transfected into PFSK-1 cells. As shown in Fig 2B, parental MCVSyn genomes expressed viral miRNA moieties that were readily detectable by northern blotting at 4 days post infection. As expected, no mcv-mir-M1 pre- or mature miRNAs were produced in cells transfected with MCVSyn-hpko mutants. As shown in Fig 2C, the absence of viral miRNA expression resulted in substantially higher expression of LT-Ag on the protein level. To investigate whether elevated LT-Ag expression also affected the efficacy of viral DNA replication, we performed a *DpnI* resistance assay on HIRT extracts. As shown in the Southern Blots of Fig 2D, MCVSyn-hpko genomes indeed replicated to appreciably higher levels when compared to the wt genome. Collectively, the above data thus suggest that i) complementary target sites in full length LT-Ag transcripts are accessible to mcv-mir-M1 binding, ii) levels of mcv-mir-M1 expressed by replicating MCPyV genomes are sufficient to induce substantial downregulation of LT-Ag expression and iii) the extent of LT-Ag downregulation mediated by mcv-mir-M1 is sufficient to limit the replication of transfected MCPyV genomes.

### Identification of transcriptional initiation and polyadenylation sites

The use of our semi-permissive replication system extended the possibility to perform an in depth analysis of transcripts expressed by intact viral episomes. In addition to providing valuable information about the structure of coding transcripts, we expected that such analyses would also provide clues with regard to the mechanisms that control viral miRNA expression. As MCPyV transcripts have thus far only been evaluated by Northern Blotting in cells transfected with early region expression cassettes driven by a heterologous CMV promoter [8], the location of transcriptional initiation and polyadenylation sites remains unknown. Since such sites are often difficult to capture in standard RNA-seq protocols due to the usually poor coverage of accurate 5'- and 3' transcript ends, we performed 5'- and 3'-RACE on RNA isolated from MCVSyn transfected PFSK-1 cells after 4 days of transfection.

**Fig 3:**
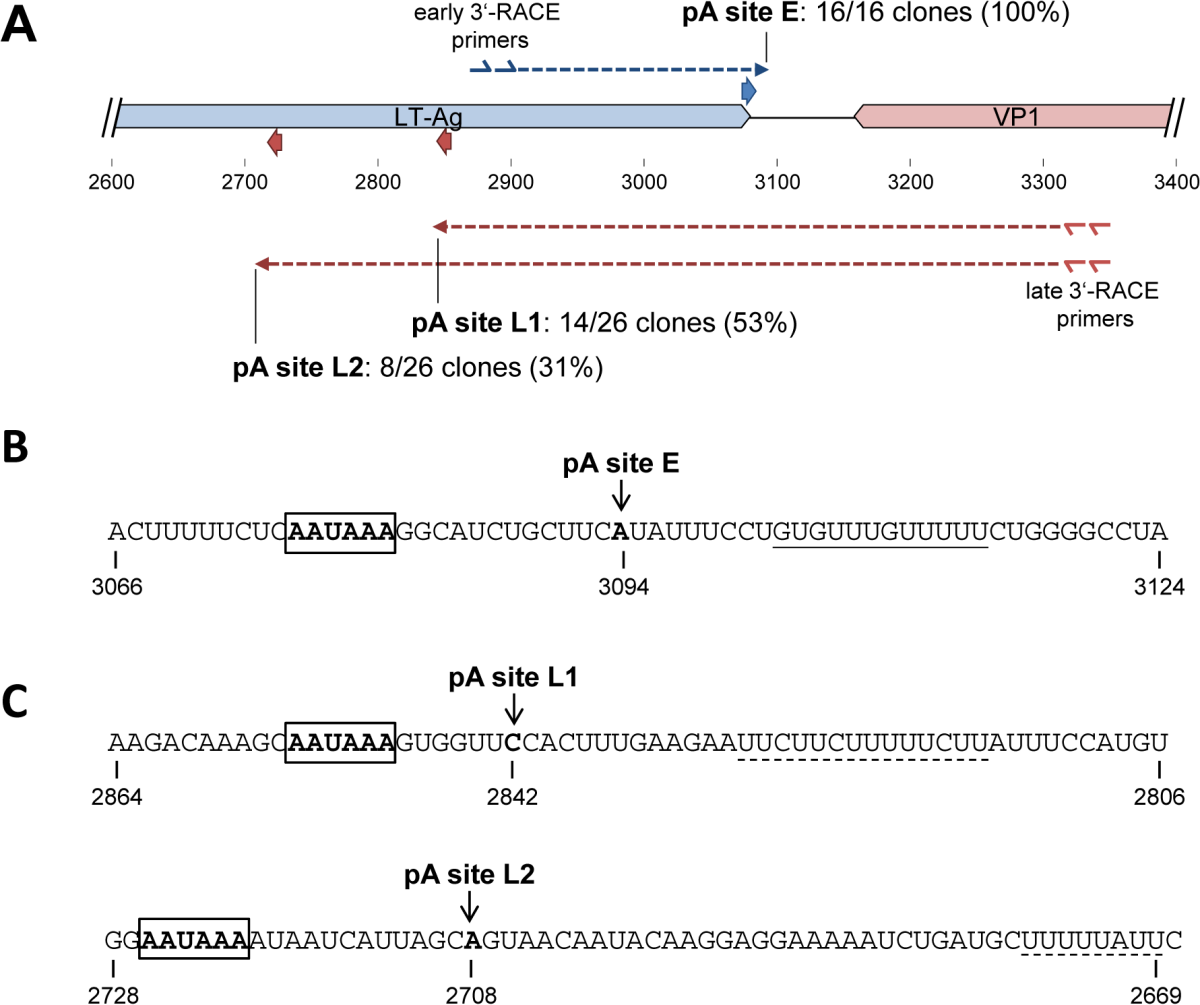
Mapping of early and late strand polyadenylation sites. (**A**) Schematic representation of early (blue) and late (red) strand polyadenylation sites as identified via 3’-RACE analysis. The location of nested gene specific primers is indicated by double arrows. Dashed arrows indicate individual clones recovered during 3’-RACE analysis for each of the strands. (**B, C**): Sequence representation of the identified polyadenylation on the early (**B**) and late (**C**) strands. The nucleotide after which cleavage occurs is marked by an arrow. Canonical polyadenlyation signals upstream of the cleavage sites are boxed. GU- or U-rich regions downstream of the cleavage sites are underlined with solid or dashed lines, respectively.

Polyadenylation sites were determined with a conventional 3’RACE protocol, using gene specific 5'-primers for the distal coding regions of early and late transcripts together with anchored oligo dT 3'-primers (Fig 3A). Amplification products were subcloned in bulk, and between 16 and 26 (for early and late transcripts, respectively) randomly picked clones were subjected to Sanger sequencing. As shown in Fig 3A and 3B, 100% (16 of 16) clones derived from early transcripts terminated at position 3094, 14 nucleotides downstream of a canonical polyadenylation signal (AAUAAA) which overlaps with the T-Ag stop codon, and 9 nucleotides upstream of a GU-rich element (Fig 3B). The 3’-RACE products from late transcripts were more diverse: As shown in Fig 3A and 3C, 14 (53%) of the 26 clones terminated at position 2842 (pA site L1), 317 nucleotides downstream of the VP1 stop codon. Another 8 clones (31%) terminated in a distance of 451 from the VP1 stop codon at position 2708 (pA site L2). Canonical polyadenylation signals were observed immediately upstream of both cleavage sites (Fig 3C). Although U-rich regions are present 13 or 31 nucleotides downstream of pA sites L1 and L2, respectively, neither site exhibits a clearly discernible GU-rich element. The four remaining clones from late transcripts were predominantly found at A-rich regions of the viral genome, suggesting they had resulted from mispriming of the oligo dT primers to internal regions of viral transcripts extending into the early region. Together, these results suggested highly efficient termination of early transcripts, but relatively weak late polyadenylation signals that allow at least some transcriptional read-through.

**Fig 4:**
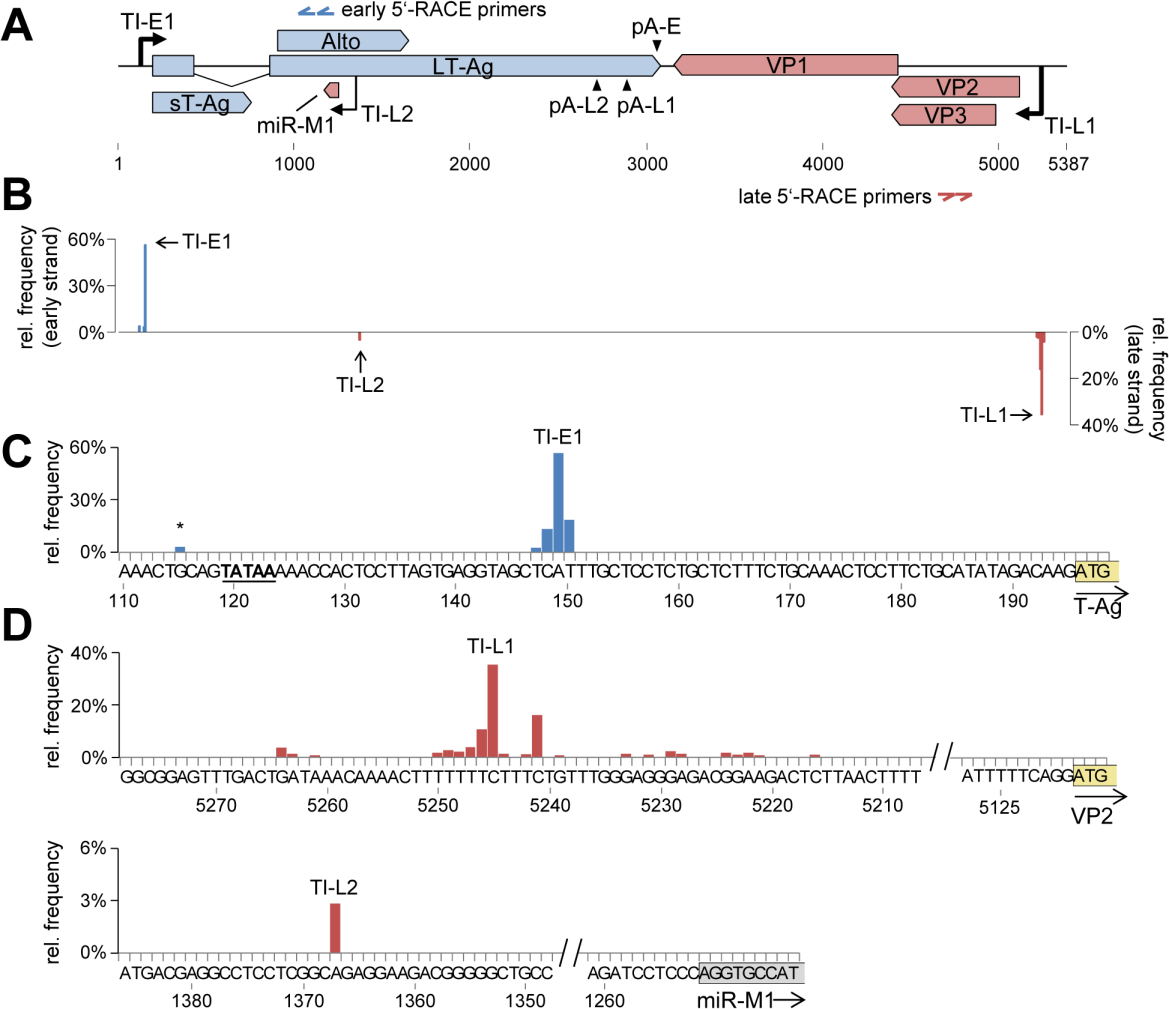
Mapping of early and late strand transcriptional initiation sites. (**A**) Schematic depiction of the MCPyV genome. The binding sites of nested gene specific 5’-RACE primers for early and late strand are indicated by blue or red double arrows, respectively. The identified transcriptional initiation zones TI-E1 (early strand) or TI-L1 and –L2 (late strand) are shown as black arrows drawn towards the top or bottom, respectively. (**B**) Relative frequency of transcriptional initiation site (TIS) reads on the early (positive axis, blue) or late (negative axis, red) strands. (**C**) Detailed depiction of TIS coverage near the major early initiation zone TI-E1. A TATA box located upstream of TI-E1 is underlined. A minor initiation site upstream of the TATA box is marked with an asterisk. The T-Ag start codon is boxed and highlighted in yellow. (**D**) Depiction of TIS read coverage near the major late initiation zone TI-L1 (top panel), and a second initiation site outside of the NCCR (TI-L2, lower panel). The VP2 start codon is boxed and the first 9 nucleotides of the genomic mcv-miR-M1 locus are boxed and highlighted in yellow or grey, respectively.

For the determination of transcriptional initiation sites we employed Cap-dependent 5'-RACE, a protocol which greatly decreases the rate of false positives that result from degradation products and/or premature termination of reverse transcription. Gene-specific RT-PCR anchor primer sites for late transcripts were situated approximately 400 nucleotides downstream of the VP2 start codon (Fig 4A). To allow the detection of putative transcripts that may initiate near the recently identified ALTO reading frame [10], primers for early transcripts were designed to bind to a region in the second exon of the LT-Ag. As we expected that initiation sites may be more heterogeneous than polyadenylation sites, amplification products were analyzed by high throughput sequencing (HTS) instead of Sanger sequencing. After mapping of reads to the MCPyV genome, we counted the number of reads that initiated at a given nucleotide position. Only nucleotides which received at least 1% of the total reads were considered as potential transcriptional initiation sites.

As shown in Fig 4B and 4C, the great majority (93%) of the ~55.000 analyzed reads from early transcripts initiated between nucleotides 147–150 (TI-E1 in Fig 4B and 4C) with a marked peak at position 149 (56.7% of all reads). As shown in Fig 4C, a canonical TATA Box is present 26 nucleotides upstream of the major initiation site. A second, much weaker accumulation of reads (4%) was observed between nucleotides 112 and 120, with a peak at nucleotide position 115 (marked with an asterisk in Fig 4C), suggesting that a minority of early transcripts initiates upstream of the TATA box. Of note, approximately 10% of the corresponding reads exhibited a splice junction which fused nucleotide 141 to the previously identified splice acceptor of the second LT-Ag exon. This splice event generates a transcript in which the first AUG triplet is the start codon of the ALTO open reading frame, 49 nucleotides downstream of the transcript's 5'-end. While we have formally confirmed the existence of the junction by RT-PCR primers (S2 Fig, lane 3), whether or not such rare transcripts contribute to the production of ALTO remains to be established. The remainder of reads was randomly scattered across the viral genome, indicating they were derived from breakdown or premature RT termination products.

Consistent with the fact that the region between the origin of replication and VP2 lacks a canonical TATA box, we observed that late transcripts were derived from a broader initiation zone (termed TI-L1 in the following) located between nucleotides 5264 and 5222, with a total of 15 nucleotide positions accumulating at least 1% of the ~159.000 total reads (Fig 4B and upper panel in Fig 4D, S4 Dataset). The bulk of initiation sites (~72%) mapped to a C/T-rich region between nucleotides 5241 and 5250, with the major initiation site (35% of all reads) being located at position 5245, 127 basepairs upstream of the VP2 start codon. Interestingly, another 4.452 reads (2.8%) mapped to nucleotide position 1367, well outside of the NCCR (TI-L2 in Fig 4B and lower panel in Fig 4D). The observed initiation site is located 116 nucleotides upstream of the mcv-miR-M1 locus, suggesting the existence of miRNA-encoding transcripts that originate outside of the NCCR.

### mcv-miR-m1 can be expressed independently of NCCR-initiated transcription

**Fig 5:**
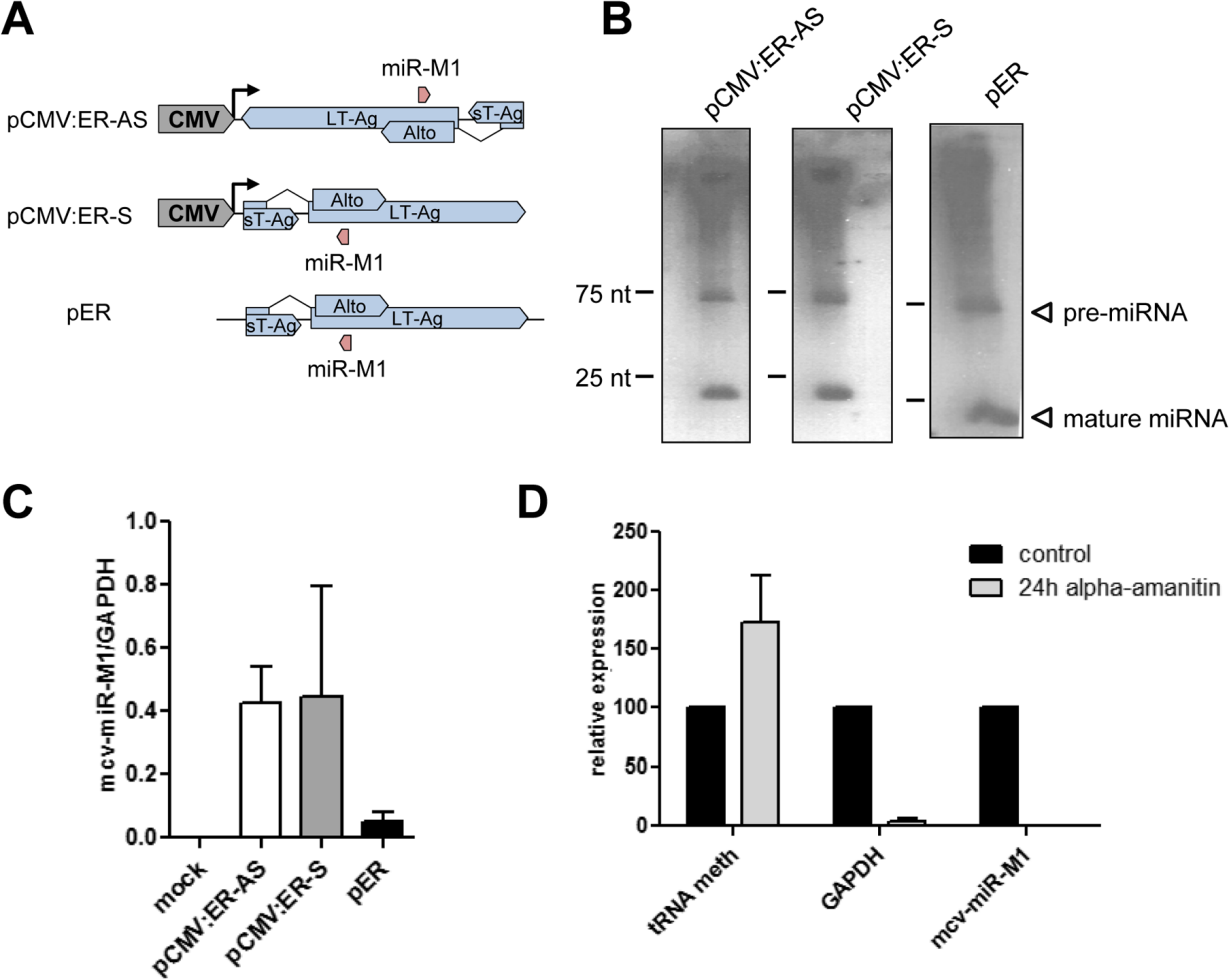
mcv-miR-M1 can be expressed independently of NCCR-initiated late gene expression. **(A)** Schematic illustration of heterologous mcv-miR-M1 constructs. pCMV:ER-AS and-S contain the entire early coding region in antisense (ER-AS) or sense orientation (ER-S) relative to the CMV promoter. pER contains the entire early coding region without an heterologous promoter. **(B)** Small RNA Northern Blot analysis of PFSK-1 cells after 2 days of transfection with the mcv-miR-M1 constructs shown in A. Blots from cells transfected with CMV constructs were exposed overnight. The blot from pER transfected cells was exposed for five days to facilitate visualization of miRNA signals. **(C)** Quantitative stem-loop RT-PCR for mcv-miR-M1-5p expression in PFSK-1 cells after 2 days of transfection with the mcv-miR-M1 constructs shown in A. **(D)** Quantitative RT-PCR for mcv-miR-M1-5p (right columns), GAPDH mRNA (center columns) or tRNA meth expression (left columns) in PFSK-1 cells after 2 days of transfection with construct pER. Expression of the indicated transcripts in the presence of α-amanitin (light grey bars) is displayed as mean values from three independent experiments relative to the expression in untreated control cells (black bars).

Given the observation of transcripts initiating upstream of the viral miRNA we sought to investigate whether mcv-miR-M1 could be expressed independently of NCCR-initiated transcription. For this purpose, we sub-cloned the entire early T-Ag coding region in either sense or antisense orientation downstream of an heterologous CMV promoter (pCMV:ER-S and –AS, respectively; see Fig 5A). As expected, forced transcription of the early region antisense strand gave rise to readily detectable pre- and mature miRNA moieties of mcv-miR-M1 (Fig 5B, left panel). However, similar levels of miRNA expression were observed when the CMV-promoter initiated transcription traversed the early region in the sense (i.e. T-Ag coding) orientation (Fig 5B, center panel). Indeed, a promoterless construct harboring the entire early region (pER) was likewise able to express the viral miRNA (Fig 5B, right panel), albeit at considerably (approx. 10 fold) lower levels than either CMV promoter-driven construct (see GAPDH-normalized stem-loop RT-qPCR data in Fig 5C; note that the Northern Blot in the right panel Fig 5B was exposed for longer time period than those shown for the pCMV constructs). While we presently cannot explain the seemingly disparate observation that strong miRNA expression was observed independent of the CMV promoter’s orientation relative to mcv-miR-M1, we suspect that CMV-promoter driven transcription through the locus may activate an intrinsic promoter. The fact that we had observed a transcriptional initiation site approx. 100 nt. upstream of the miRNA using a 5’-CAP dependent RACE protocol suggested that such transcripts are likely produced by RNA polymerase II. To investigate this assumption, we treated pER-transfected PFSK-1 cells with α-amanitin, a potent inhibitor of RNA-polymerase (RNA-pol) II, and investigated mcv-miR-M1 expression 24 hours later. As controls for RNA pol II and III transcribed RNAs, we additionally measured levels of GAPDH mRNA and tRNA-meth, respectively. As shown in Fig 5D, α-amanitin treatment strongly reduced expression of GAPDH and mcv-miR-M1, but not that of tRNA-meth. Hence, an intrinsic promoter activity within in the early region of MCPyV can lead to RNA pol II-dependent transcription of mcv-miR-M1.

### The NCCR and a region upstream of the mcv-miR-M1 locus are highly enriched for activation-associated histone marks

**Fig 6:**
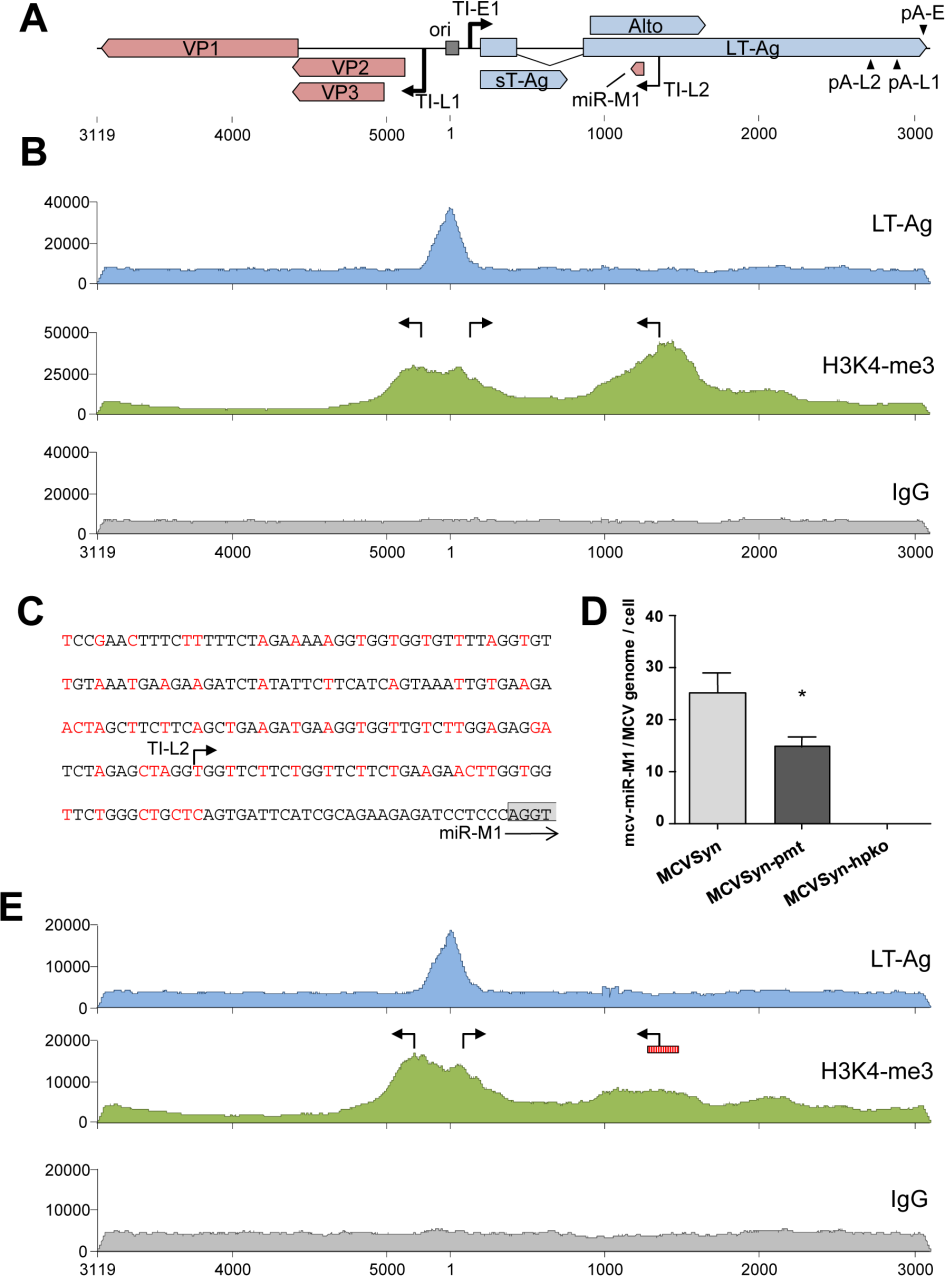
ChIP-seq analysis and miRNA expression in wt and mutated MCVSyn genomes. (**A**) Schematic depiction of the MCPyV genome with polyadenylation and transcriptional initiation sites as identified by RACE analyses. The position of the viral core origin of replication (ori) is marked by a grey box. (**B, E**) ChIP-seq profiles of LT-Ag (top panel, blue), H3K4me3 (center panel, green) or the negative IgG control (bottom panel, grey) along the MCPyV genome in PFSK-1 cells after 4 days of transfection with MCVSyn (**B**) or MCVSyn-pmt (**E**). Positions of early and late transcriptional initiation sites are marked by bent arrows pointing right and left, respectively. The putative promoter region mutated in MCVSyn-pmt is symbolized by a vertically hatched box in E. Graphs depict raw ChIP-seq read coverage; note that absolute read numbers depend on the efficiency of individual immunoprecipitations and thus only the relative coverage distribution along the viral genome is meaningful. (**C**) Mutations in the putative promoter region upstream of the mcv-miR-M1 locus. Substituted nucleotides are shown in red. The positions of the transcriptional initiation site TI-L2 is indicated by an arrow. The first four nucleotides of the mcv-miR-M1 locus are boxed in gray. **(D)** Quantitative stem-loop RT-PCR evaluation of mcv-miR-M1-5p expression in PFSK-1 cells after 4 days of transfection with MCVSyn (left), MCVSyn-pmt (center), or MCVSyn-hpko (negative control, right). Expression levels were normalized to the number of MCVSyn genomes per cell as determined by qPCR from genomic DNA. Mean values and standard deviations were calculated from three independent experiments. mcv-miR-M1 expression in MCVSyn-pmt is significantly decreased in comparison to MCVSyn (unpaired t-test).

If the region upstream of the mcv-miR-M1 locus exhibits promoter activity, then intact episome should exhibit an open chromatin conformation that permits transcriptional initiation at this position. To investigate this notion, we performed ChIP-seq experiments to evaluate patterns of the postranslational histone modification H3K4me3, a mark which is strongly enriched at transcriptional start sites. Additionally, we performed ChIP-seq to elucidate binding patterns of LT-Ag binding across the viral episome. For this purpose, we transfected PFSK-1 cells with MCVSyn and, 48 hours later, performed chromatin immunoprecipitation with antibodies specific for H3K4me3, or with the LT-Ag antibody CM2B4. An immunoprecipitation with IgG served as a negative control. The complete coverage data is given in S5 Dataset. As shown in the top panel of Fig 6B, the CM2B4 antibody produced a marked peak centered at the core origin of replication, consistent with the previously observed binding of LT-Ag to an array of GRGCC pentamers located in this region [41, 42]. No additional peaks were observed, indicating that, at least under the conditions used here, LT-Ag appears not to stringently bind to other loci on the viral episome. As expected, the NCCR of MCPyV was also highly enriched in the activation-associated histone mark H3K4me3 (Fig 6B, center panel). The H3K4me3 profile in this region presented as a broad peak which extended from the late to the early transcription initiation sites mapped during our 5’-RACE analysis. Indeed, consistent with our previous experiments that had suggested promoter activity of the region upstream of the viral miRNA locus, a second prominent H3K4me3-enriched zone was located within the T-Ag coding region. The summit of this peak mapped precisely to the transcriptional start site upstream of the viral miRNA locus identified during our 5’-RACE analysis. We next sought to determine whether mutation of upstream sequences would negatively affect miRNA expression. Given that the H3K4me3 enriched region is located in the early coding region, deletion of the putative promoter would also disrupt LT-Ag expression and thus abrogate the replication ability of MCVSyn episomes. However, we hypothesized that introduction of synonymous triplet mutations which preserve the LT-Ag coding capacity may be sufficient to ablate or reduce promoter activity. Accordingly, we generated a MCVSyn mutant (termed MCVSyn-pmt in the following) with a total of 68 triplet mutations in a ~200 bp region located 28 nt upstream of the miRNA locus (Fig 6C). Introduction of the same set of mutations in the context of early region-only construct pER (see Fig 5A) and subsequent transfection of the resulting plasmid pER-pmt into PFSK-1 cells confirmed that the mutations led to a significant reduction of NCCR-independent miRNA expression (S3 Fig).

To investigate the effect of the mutations on miRNA expression by full length genomes, MCVSyn-pmt or the parental MCVSyn construct were transfected into PFSK-1 cells, and levels of mature mcv-miR-M1-5p were evaluated 48h later by quantitative stem-loop PCR. The hairpin knockout mutant MCVSyn-hpko served as a negative control. As shown in Fig 6D, MCVSyn-pmt indeed expressed mcv-miR-M1-5p at significantly lower levels compared to the wildtype genome, albeit the residual expression levels (approx. 60%) were higher than those observed with the early region construct pER-pmt (approx. 25%; see S3 Fig).

To investigate the effect of the introduced mutations on the chromatin level, we additionally performed ChIP-seq analysis of PFSK-1 cells transfected with MCVSyn-pmt. As expected, neither LT-Ag binding to the viral origin nor H3K4me3 accumulation at the NCCR was affected in MCVSyn-pmt. However, in accord with the observed decrease in mcv-miR-M1-5p expression levels, the mutations resulted in the almost complete elimination of the H3K4me3 peak upstream of the miRNA locus. Collectively, the above data thus suggest that the genomic region upstream of mcv-miR-M1 exhibits promoter activity and can contribute to NCCR-independent expression of the viral miRNA in the context of replicating episomes. However, given that elimination of the H3K4m3 peak had reduced but not abrogated miRNA expression, we suspected that a considerable fraction of the viral miRNA may also be generated from late strand transcripts that originate from the NCCR and ignore the apparently weak late strand polyadenylation sites. To investigate this possibility, and to furthermore evaluate the overall structure of viral transcripts and the influence of the viral miRNA on transcript abundance, we proceeded to perform strand-specific mRNA-seq experiments.

### RNA-seq reveals profound transcriptional read-through of late transcripts and suggests that mcv-miR-M1 cleaves early transcripts

**Fig 7:**
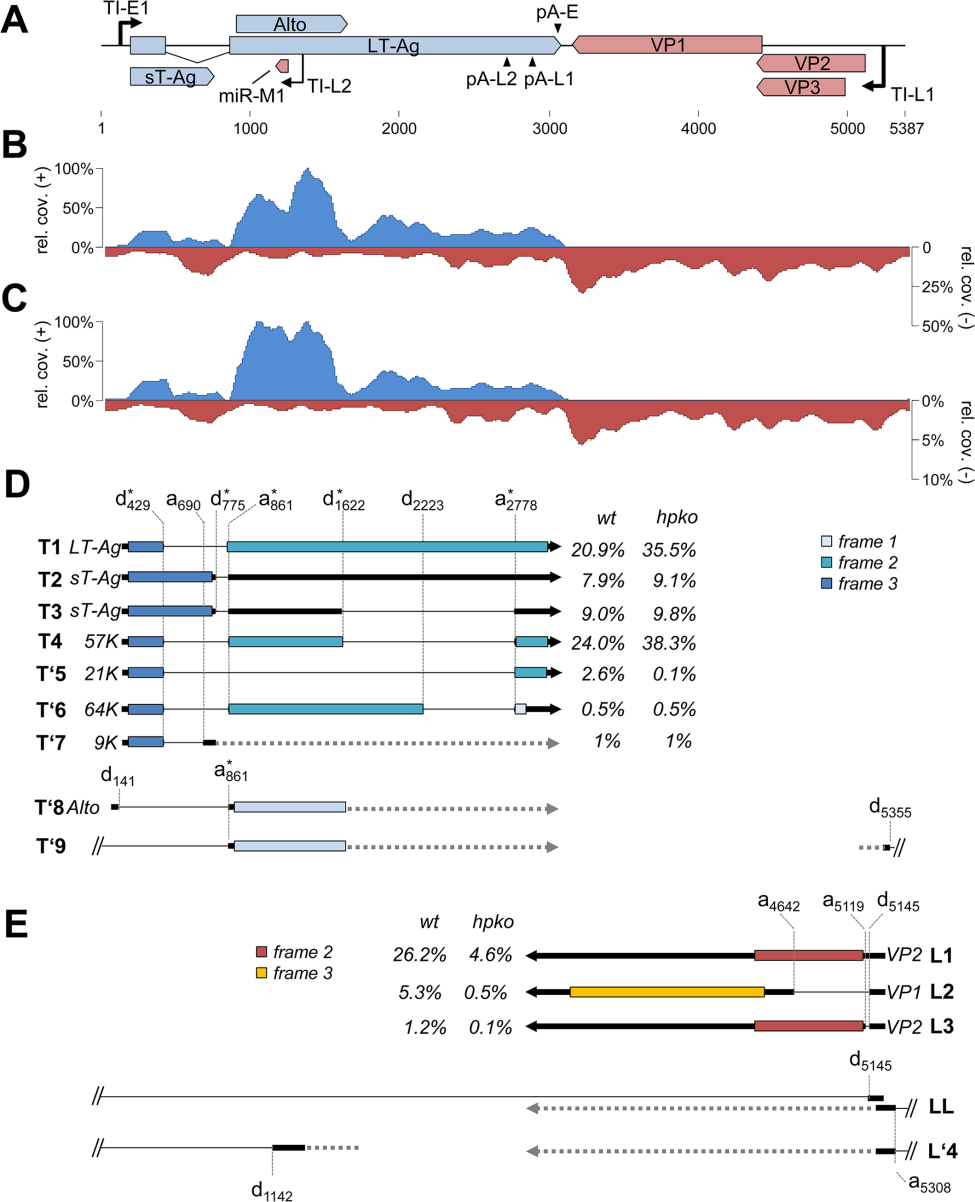
RNA-seq analysis of replicating MCPyV genomes. (**A**) Schematic depiction of the MCPyV genome. **(B, C)** RNA-seq coverage of MCVSyn (B) or MCVSyn-hpko (C) genomes in PFSK-1 cells after 4 days of transfection. Read coverage on the early (positive axis; blue) or late strand (negative axis; red) is shown relative to the maximally observed nucleotide coverage (set to 100%). Note that the negative/late strand axis is shown at a lower scale in C to facilitate comparison of late strand read coverage profiles. **(D, E)** Structure of known or predicted early (D) or late (E) transcripts. The position of donor or acceptor sites is indicated by vertically drawn hatched lines. Previously identified donors or acceptors are marked with asterisks. Known or predicted protein products and estimated abundance values are given next to all transcripts that map to the major early or late transcription cassettes (T1-T’7 and L1-L3, respectively). We did not estimate abundance for transcripts that contain splice junctions with donors or acceptors located outside of the major transcriptional units (T’8, T’9, LL, L’4). For simplicity, regions of transcripts which are of unknown structure, or that may display splice patterns which are of no immediate consequence for coding potential are symbolized by horizontal hatched lines. Coding regions (first AUG-initiated ORF) in each of the transcripts are shown by boxes that are colored according to reading frame relative to the genomic position of major early or late transcriptional start positions (149 and 5245, respectively).

To evaluate viral transcription patterns we transfected PFSK-1 cells with MCVSyn or the miRNA knockout MCVSyn-hpko and harvested mRNA after 4 days of transfection. Two independent rounds of transfection and sequencing were carried out for each the parental and mutant genomes. In Fig 7B and 7C, we present coverage plots which represent the accumulated/mean data of the replicates. S4 Fig shows the individual data plots for each of the replicates and demonstrates that both experiments produced near-identical results. Full coverage data are provided in S6 Dataset.

Consistent with the observation of a highly efficient early polyadenylation site we observed that the coverage of early transcripts exhibited a sharp decline towards the 3'-end of T-Ag coding sequences. As shown in the upper plot in Fig 7B and Table 3, in MCVSyn transfected cells greater than 99% of all reads from the early strand mapped to the region flanked by the transcriptional initiation and polyadenylation sites identified in our RACE analysis. In contrast, the coverage data for transcripts originating from the late strand were indicative of profound read-through beyond polyadenylation sites pA-L1 and-L2. While approx. 60% of late strand reads mapped to the region delineated by the initiation site TS-L1 and the late polyadenylation sites, the remaining 40% were derived from the antisense strand of the early region and the NCCR. As shown in Fig 7C, the overall late strand coverage profiles in MCVSyn-hpko transfected profiles were near identical to those observed in cells transfected with the parental genome. However, consistent with the observation that mcv-miR-M1 negatively regulates LT-Ag expression, the relative fraction of reads derived from early transcripts was considerably increased in MCVSyn-hpko transfected cells (90% in MCVSyn-hpko vs. 61% in MCVSyn transfected cells; see Table 3). Moreover, early strand coverage profiles in PFSK-1 MCVSyn-hpko cells showed increased coverage immediately downstream of the region antisense to mcv-miR-M1. This is consistent with the fact that the RNA-seq protocol captures polyadenylated RNAs and therefore selects for 3’-fragments of miRNA-cleaved transcripts. In contrast, 5’-cleavage products lack a polyA tail and thus are lost prior to library preparation, resulting in a relative decrease in read counts upstream of the cleavage site. Together, the above data thus suggest that viral MCPyV miRNAs negatively regulate T-Ag expression via cleavage of early strand transcripts.

**Table 3:** RNA-seq summary statistics.

reads	MCVSyn	MCVSyn-hpko
total reads	486,144,182	439,827,868
mapped to MCPyV:	114,326	84,527
- early strand	69,709	76,060
- major early transcription cassette^**a**^	69,019	75,552
- late strand	44,617	8,467
- major late transcription cassette^**b**^	27,504	5,176

***a*, *b***: number of reads mapped to regions delineated by transcriptional start and polyadenylation sites identified in this study (nts. 149 to 3094 (+) and 2708 to 5245 (-), respectively).

### RNA-seq identifies novel splice products from early and late transcripts

Thus far, elucidation of viral splice patterns has been limited to evaluation of the ectopically expressed early region [8]. To investigate splice patterns of early and late transcripts expressed from full-length genomes, we analyzed the structure and frequency of spliced reads from our RNA-seq experiments. We considered such reads as evidence of an authentic splice event if i) the event was supported by at least two independent observations among the individual experiments, ii) the junction was consistently observed in both replicates of MCVSyn or MCVSyn-hpko transfections and iii) the splice sites exhibited the sequence features commonly observed at donor and acceptor sites. For each of the identified sites, we additionally calculated the number of unspliced reads to evaluate the efficiency with which the given site underwent splicing. In Table 4, we present the identified donor sites and junctions along with the read numbers and splice frequencies as calculated from the accumulated data from both datasets of MCVSyn or MCVSyn-hpko transfected cells. The structures of known or novel early and late transcripts are shown in Fig 7D and 7E, respectively. For transcripts mapping to the major early and late transcription cassettes, an estimation of relative abundance is shown after each transcript. S5 Fig shows the sequence context of known and the novel splice sites observed in this study.

**Table 4:** Splice junctions observed in MCVSyn- and MCVSyn-hpko-transfected cells.

			MCVSyn		MCVSyn-hpko	
strand	donor	acceptor ^a^	reads	% donor reads	reads	% donor reads
+	d_141_	n.s.	136	86.62%	146	74.49%
		a_861_	21	13.38%	50	25.51%
+	d_429_	n.s.	512	27.47%	478	20.46%
		a_861_	1,273	68.29%	1,832	78.42%
		a_690_	31	1.66%	24	1.03%
		a_2778_	48	2.58%	2	0.09%
+	d_775_	n.s.	47	6.39%	17	1.96%
		a_861_	688	93.61%	849	98.04%
+	d_1622_	n.s.	560	47.10%	464	48.43%
		a_2778_	629	52.90%	494	51.57%
+	d_2223_	n.s.	1,502	97.79%	1,347	98.54%
		a_2778_	34	2.21%	20	1.46%
+	d_5355_	n.s.	23	92.00%	24	75.00%
		a_861_	2	8.00%	8	25.00%
-	d_1142_	n.s.	603	94.66%	84	87.50%
		a_5308_	34	5.34%	12	12.50%
-	d_5145_	n.s.	1,110	76.18%	292	86.39%
		a_4642_	223	15.31%	31	9.17%
		a_5308_	74	5.08%	12	3.55%
		a_5119_	50	3.43%	3	0.89%

***a***: ‘n.s.’ denotes non-spliced reads extending over the given donor positions.

The great majority of splice events among early transcript mapped to the junctions previously identified by Shuda and colleagues [8]. Together, these transcripts (T1 to T4 in Fig 7D) are estimated to account for approximately 95% of all early strand mRNAs. Additional splice junctions were detected only at low frequency. The corresponding putative mRNAs include transcripts (tentatively named T’5 through T’7), which are predicted to encode T antigens with estimated molecular weights of 9, 21 or 64 kDa. All of these protein products contain the first 93 amino acids shared by LT- and sT-Ag, but entirely or partially lack the sequences encoded by the second exon of LT-Ag. Similar low early region transcripts of low abundance have been previously observed in other polyomaviruses, but in most cases it is not clear whether their protein products are of biological significance [43–49].

While splice events that originated outside of the major early transcription cassette were infrequent, the majority of such events consisted of the junction already observed during our 5’-RACE analysis in the putative ALTO-encoding transcript (d141-a861 in Table 4, transcript T’8 in Fig 7E). Additionally, we observed a very rare splice event, which uses the same acceptor at position 861, but a donor upstream of the origin (d5355). Such transcripts may either be produced by upstream initiation events which were too infrequent to be picked up by our RACE analysis, or by occasional read through beyond the early region polyadenylation site. RT-PCR formally confirmed the existence of this rare splice event (S2 Fig, lane 1).

Among the late strand transcripts we detected a total of 5 splice junctions involving 2 donor and 3 acceptor sites (Table 4 and Fig 7E). Abundance estimation predicts that the majority of late messages are unspliced transcripts which encode VP2 (L1 in Fig 7E). Splicing from a donor at position 5145 to an acceptor 503 nt downstream (a5119) in approximately 10–15% of late strand transcripts generates a message in which the first AUG codon initiates the VP1 ORF (transcript L2). The same donor is joined to an alternative acceptor at position 5119 in another 2–4% of late transcripts (L3 in Fig 7E). This event leads to removal of an immediately upstream of the VP2 start codon, and the resulting L3 transcripts are thus predicted to code for VP2. We did not detect transcripts which splice to the start codon of the predicted VP3 ORF.

Interestingly, 4–5% of all splice events observed for the donor at position 5145 (Table 4) connect to an acceptor (a5308) which is located upstream of the late transcriptional start sites identified in our 5’-RACE analysis (LL in Fig 7E). This splice event consequently requires primary transcripts which traverse the entire episome, similar to the leader-to-leader splice observed in other polyomaviruses [50–55]. RT-PCR analysis with junction spanning primers indicates that multiple copies of the leader can be present at the 5’-end of late transcripts (S2 Fig, lane 7), indicating that the RNA polymerase can complete several rounds of transcription along the viral episome. As the leader sequence does not contain putative AUG start codons, its presence is not expected to alter the coding capacity of transcripts L1, L2 or L3.

We additionally detected another splice event which extended over the NCCR, joining a donor at downstream of the viral miRNA (d1142) to the acceptor at position 5308. While the existence of this splice was confirmed by RT-PCR (S2 Fig, lane 5), only ~5–13% of all reads traversing the donor are spliced (Table 4), and transcripts containing this splice (tentatively named L’4 in Fig 7E) are therefore expected to be rare. As such transcripts may originate from transcriptional read-through beyond late polyadenylation sites or from transcripts which are initiated upstream of the viral miRNA, their coding potential remains unknown.

### A MCPyV miRNA knockout mutant is impaired in its ability to establish long term persistence

**Fig 8:**
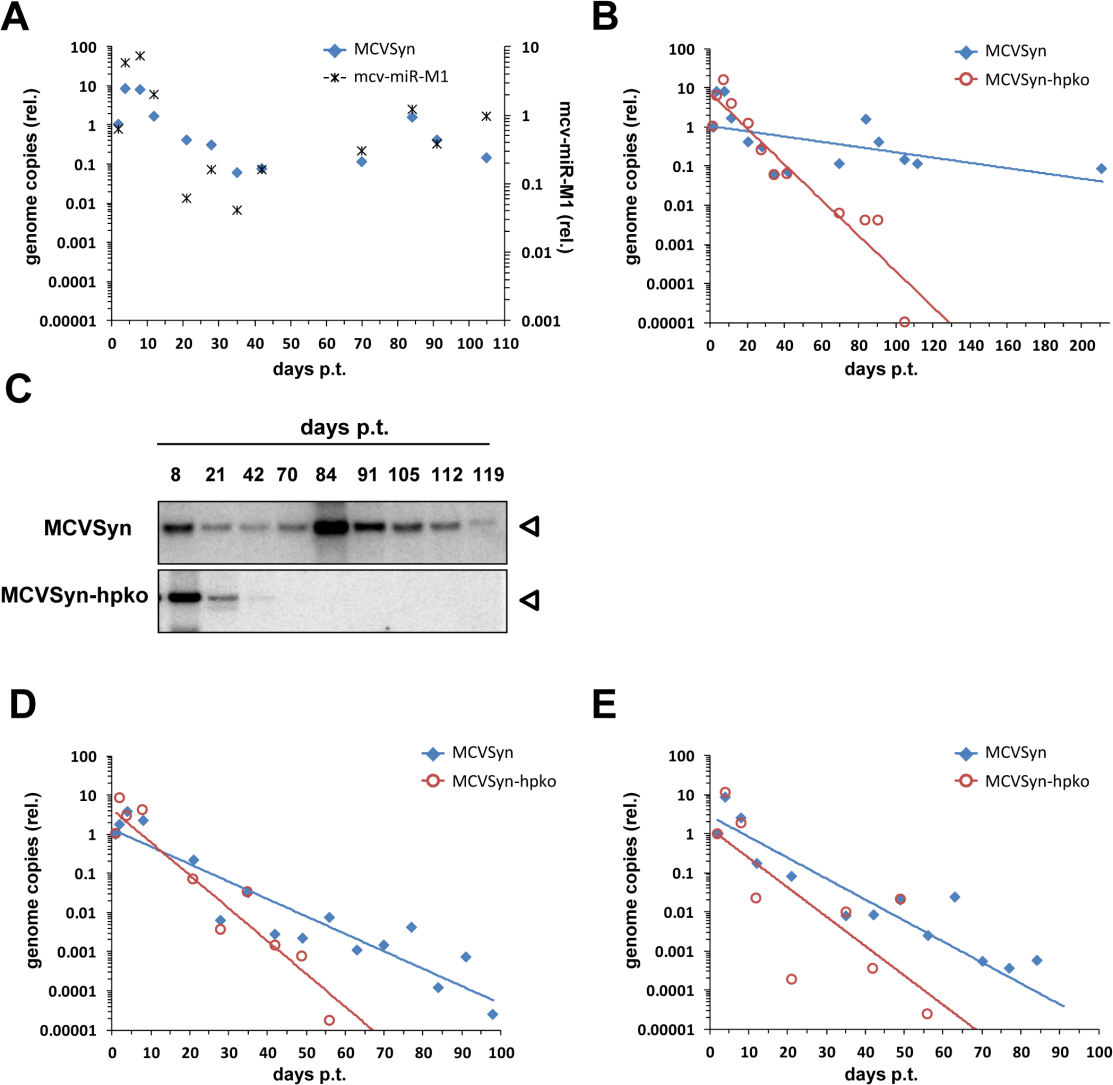
A mcv-miR-M1 knockout mutant is impaired in long-term persistence. **(A)** Normalized MCVSyn copy numbers (relative to day 2; blue filled diamonds, left axis) or mcv-miR-M1 expression levels (black asterisks, right axis) as measured by qPCR from genomic DNA or stem-loop RT-PCR of long-term PFSK-1 cultures transfected with MCVSyn. All values were normalized to genomic GAPDH copy numbers; note that values thus will reflect changes in the overall percentage of positive cells as well as changes of episome numbers per positive cell. **(B)** GAPDH-normalized MCVSyn (blue symbols, reproduced from A) or MCVSyn-hpko (red symbols) copy numbers (relative to day 2) in long-term PFSK-1 cultures. Data points were fitted with exponential regression lines (blue or red for MCVSyn or MCVSyn-hpko, respectively) to illustrate the overall trend of copy number changes. **(C)** Southern Blot analysis for the detection of replicated viral DNA of HIRT extracts from MCVSyn (top panel) or MCVSyn-hpko (lower panel). Blots were performed with the same cultures shown in A and B. **(D** and **E)** Independent repeats of long-term genome maintenance assays in MCVSyn or MCVSyn-hpko-transfected PFSK-1 cells. The initial percentage of transfected cells in PFSK-1:MCVSyn as well as PFSK-1:MCVSyn-hpko cultures was between 2 and 3% for all three experiments shown in this figure. MCVSyn or MCVSyn-hpko-transfected cultures did not exhibit gross growth differences and were subcultured at the same intervals.

The results described thus far demonstrate that mcv-miR-M1 efficiently suppresses early gene expression and viral DNA replication between two and four days post transfection. These time points were chosen because they guarantee robust genome amplification and allow readily detectable expression of viral genes. Interestingly, however, in an independent set of experiments we had repeatedly observed that, after transfection in a number of cell lines, MCPyV genomes remained detectable for several weeks or even months by Southern Blotting and qPCR. To confirm these findings in PFSK-1 cells, and to furthermore investigate influence of the viral miRNA expression on long term persistence of MCPyV genomes, we transfected PFSK-1 cells with MCVSyn or MCVSyn-hpko and monitored the resulting cultures for a period of at least 3 months. At regular intervals, we collected total DNA, small RNA and protein to evaluate relative MCPyV genome copy numbers as well as expression of mcv-miR-M1 and LT-Ag. Fig 8A shows relative genome copy numbers and viral miRNA expression of the wt MCVSyn episome as determined by qPCR or quantitative stem-loop RT-PCR, respectively. All values were normalized for genomic GAPDH locus copy numbers and are shown relative to the earliest sampled time point at d2 post transfection (set to 1). Consistent with the previous results from our short-term DNA replication assays, MCVSyn genomes exhibited an initial increase of relative copy numbers within the first ~10 days, which was followed by a steep decline over more than two orders of magnitude in the following two weeks. After this loss phase, however, MCVSyn copy numbers did not further decline, suggesting that viral genomes had entered a state of near-stable long term maintenance. Furthermore, temporal changes of viral miRNA expression levels closely mirrored changes in relative genome copy numbers, indicating the per-genome expression levels of mcv-miR-M1 remained stable over the course of the experiment. Interestingly, and contrary to what might have been expected based on increased DNA replication in short term assays (Fig 2D), MCVSyn-hpko genomes were unable to reach a state of stable maintenance. Whereas copy numbers of parental genomes remained stable even beyond a 6 month time point, the hairpin mutant was progressively lost from the cultures such that it became undetectable by day 105 (blue and red symbols, respectively, in Fig 8B). The qPCR results were furthermore confirmed by Southern Blotting of *DpnI*-resistant DNA (Fig 8C; note that owing to the lower sensitivity of these assays MCVSyn-hpko genomes become already undetectable at day 70).

**Fig 9:**
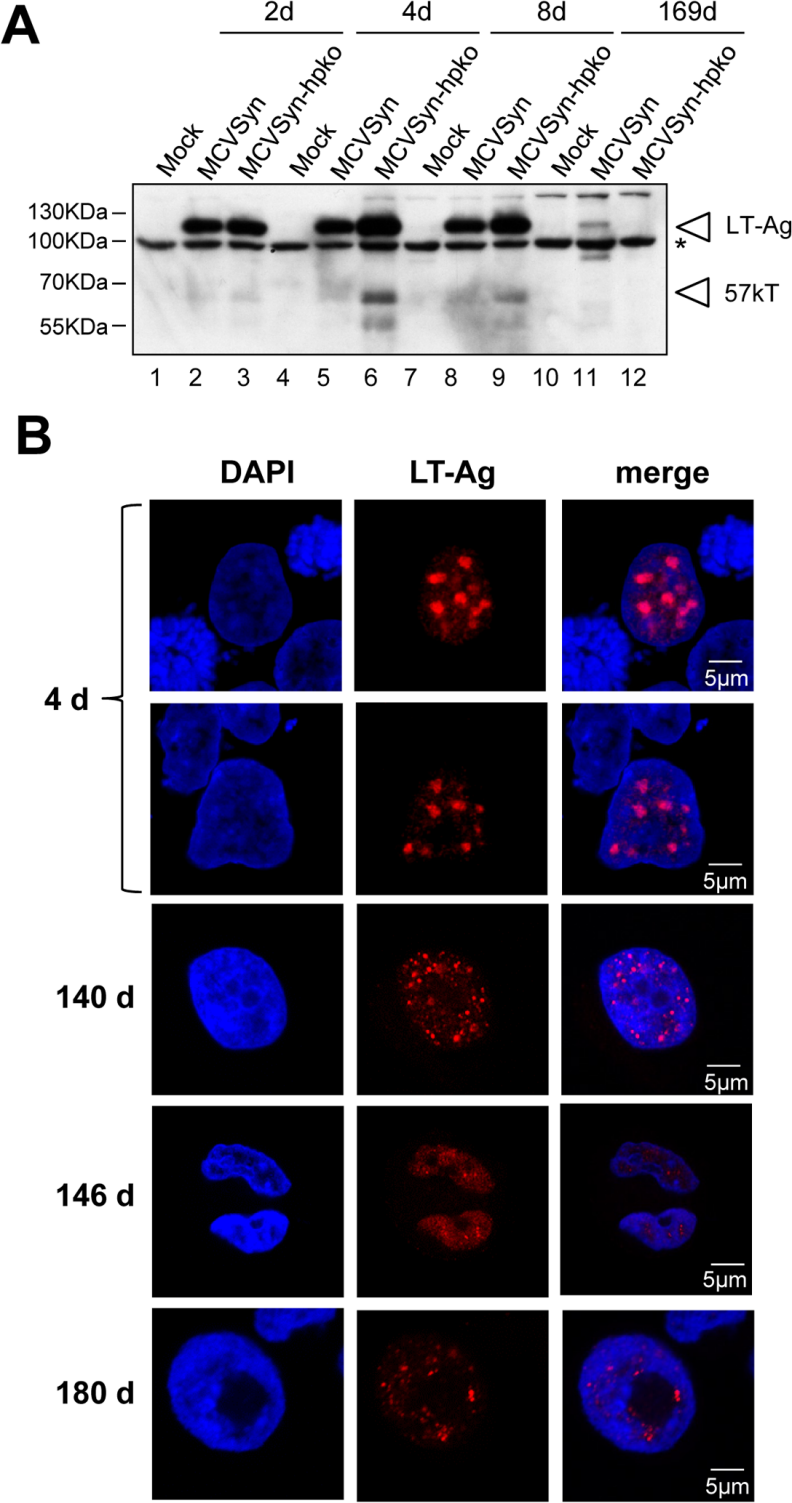
LT antigen expression in long term cultures of MCVSyn or MCVSyn-hpko transfected PFSK-1 cells. **(A)** Western blot analysis and **(B)** confocal laser scanning immunofluorescence microscopy of PFSK-1 cells transfected with MCVSyn or MCVSyn-hpko and analyzed at the indicated time points. Material was derived from the same cultures shown in Fig 8A to 8C. The asterisk in (A) denotes the position of an unspecific background band. Antibody CM2B4 recognizes LT-Ag as well as the alternative splice product 57k T. The positions of both protein bands are indicated by arrowheads.

Western Blot analysis of the bulk cultures confirmed the absence of LT antigen in MCVSyn-hpko transfected cells after the loss of genomic DNA (Fig 9A, lane 12). In contrast, LT antigen expression could be readily observed in MCVSyn transfected cultures even after more than 160 days (lane 11). To also analyze LT-Ag expression on the single cell level, we performed immunofluorescence analyses using the CM2B4 antibody. Fig 9B shows representative images from an early (4d) and several late time points of MCVSyn transfected cells. LT-Ag staining presented as distinct, strictly nuclear dots that are likely to represent foci of viral DNA replication. At 4 days post-transfection, we estimated the percentage of LT-Ag positive cells in both MCVSyn and MCVSyn-hpko transfected cultures to be approximately 2–3%. In accord with our qPCR and southern blot experiments, LT-Ag positive cells became approximately 100fold less frequent, but cells with multiple foci (albeit smaller than those observed after 4 days) remained clearly detectable for several months in MCVSyn cultures. Apart from the fact that LT-Ag positive cells were absent from late time points of MCVSyn-hpko-transfected cultures, we did not detect fundamental differences in the LT-Ag staining patterns between MCVSyn or MCVSyn-hpko transfected cells.

In Fig 8D and 8E, we show two independent repeats of our long term maintenance assays. Although parental MCVSyn genomes were less efficiently maintained in these experiments, the miRNA-deficient mutant consistently demonstrated an accelerated rate of loss and became undetectable at least four weeks earlier than the wt genome.

Although PFSK-1 cells produce only very low levels of infectious virus particles and do not allow efficient serial transmission [27], we considered it formally possible that the decreased long term persistence of MCVSyn-hpko genomes may reflect alterations in particle production. To directly investigate this scenario, we inspected viral genome copy numbers in total genomic DNA and DNaseI treated supernatants from freshly transfected PFSK1 cells (4d p.t.). In accord with our previous results, total copy numbers of viral genomes were higher in MCVSyn-hpko transfected cells (S6A Fig). However, there was no appreciable difference between DNaseI-resistant genome copy numbers in the supernatants of MCVSyn or MCVSyn-hpko transfected cells, indicating comparable levels of virion production. We additionally used freeze-thaw lysates from such cultures to inoculate fresh PFSK-1 cultures and measure the amount of nuclear viral DNA recovered after 4 or 8 days post-inoculation (S6B Fig). As expected, the overall amount of DNA recovered from infected cells was strongly reduced compared to levels observed in transfected input cultures. Again, however, we did not detect significant differences between cultures inoculated with lysates from MCVSyn or MCVSyn-hpko-transfected cells, suggesting that the differences observed in long-term maintenance assays are likely to be independent of potentially altered particle production levels.

### MCVSyn genomes persist as extrachromosomal episomes

**Fig 10:**
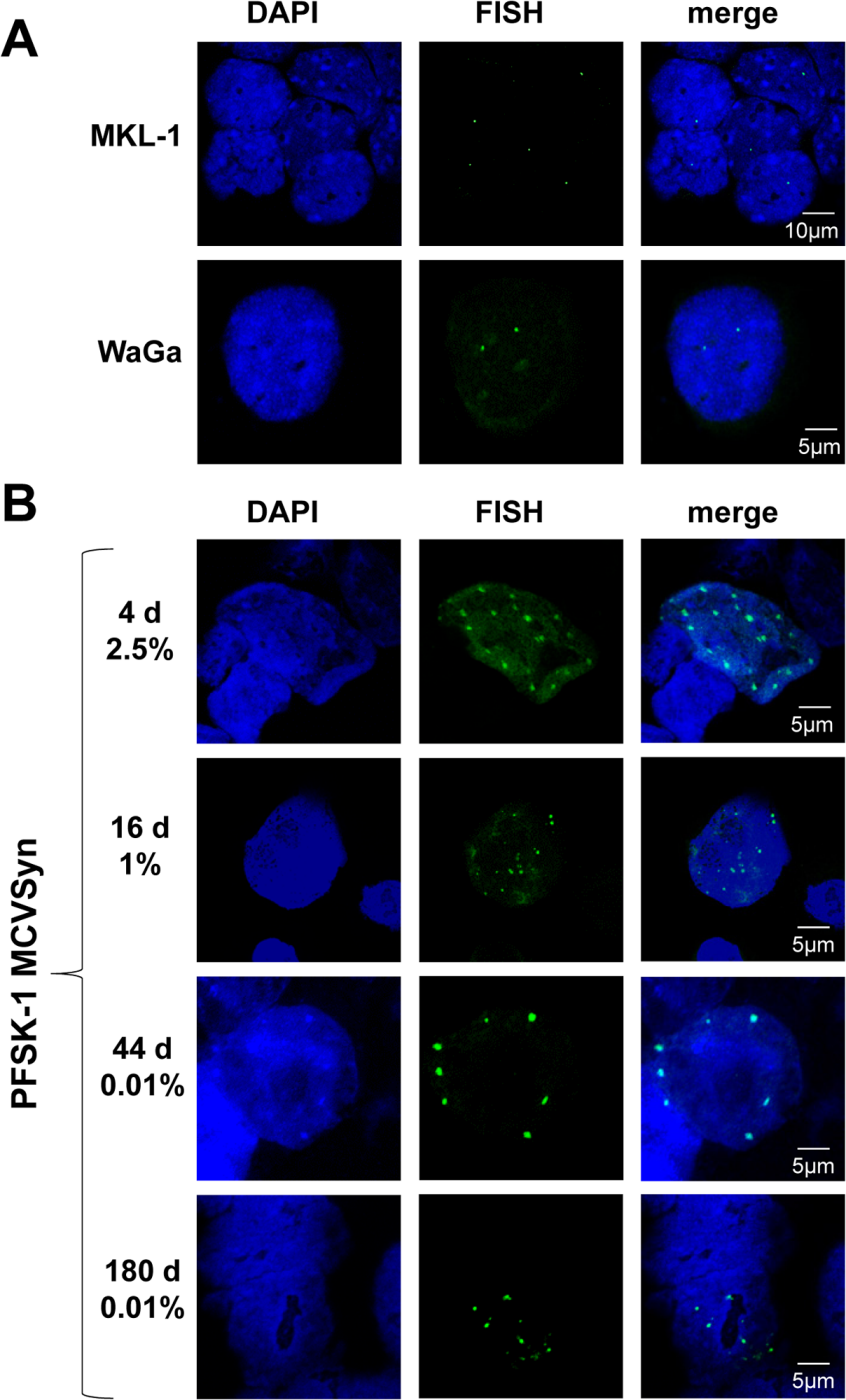
FISH analysis of MCPyV genome in MCC cell lines and long term cultures of MCVSyn transfected cells. **(A)** FISH analysis (center) and Dapi staining (left) of the MCPyV positive MCC cell lines MKL-1 and WaGa. **(B)** Analysis of MCVSyn wt transfected PFSK-1 cells at early and late time points after initial transfection. The estimated percentage of MCVSyn positive cells is indicated for each time point.

Given the very low level of infectious viral particles produced in PFSK-1 cells, we hypothesized that long term persistence of MCVSyn genomes could either result from efficient episomal maintenance, or (similar to MCC-derived cell lines) reflect stable transmission of integrated genomes. To investigate these possibilities, we first established a FISH assay for MCPyV. As a control for the sensitivity and specificity of the assay, we analyzed the two MCPyV positive MCC cell lines MKL-1 and WaGa cells. As shown in Fig 10A, we detected a single distinct signal per cell in MKL-1 and two foci in WaGa cells, indicative of one or two integration events, respectively. In contrast, FISH analysis of the long-term PFSK:MCVSyn cultures shown in Figs 8A–8C and 9 detected a considerably larger number of foci per cell nucleus (Fig 10B; see S7 Fig for exemplary images taken at a lower magnification). In accord with our LT-Ag immunofluorescence assays, while approximately 2.5% of all cells were positive for MCVSyn at 4 days post transfection, the number of positive cells dropped over time and reached a steady state of ~0.01% at late time points. In agreement with the qPCR and Southern Blotting results, we were able to detect MCVSyn positive cells by FISH for more than 160 days (lower panel in Fig 10B).

**Fig 11:**
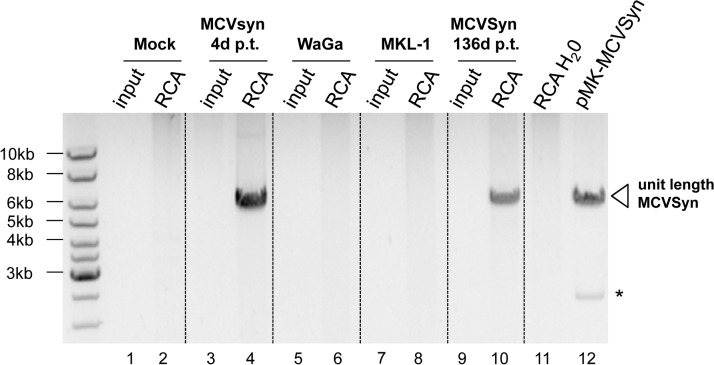
RCA analysis suggests episomal persistence of MCVSyn genomes. Inverted image of a ethidium bromide-stained agarose gel with input material (lanes 1,3,5,7 and 9) or RCA products (lanes 2,4,6,8, 10 and 11) from mock-transfected PFSK-1 cells (lanes 1 and 2), MCVSyn-transfected PFSK-1 cells at 4 days (lanes 3 and 4) or 136 days (lanes 9 and 10) post transfection, the MCC-derived cell lines WaGa or MKL-1 (lanes 5 and 6 or 7 and 8, respectively) or a water control (lane 11). DNA was subjected to restriction enzyme digestion to produce linear, unit-length viral genomes from concatameric RCA products. For size comparison, lane 12 shows unit length MCVSyn genomes excised from plasmid pMK-MCVSyn. The asterisk marks the position of the bacterial pMK plasmid backbone.

While the observation of multiple nuclear foci of viral DNA argues against rare integration events being responsible for long-term maintenance, FISH analysis cannot provide definite proof of episomal persistence. To more directly address this issue, we therefore performed rolling circle amplification (RCA) for MCPyV DNA, a protocol which selectively amplifies circular templates and produces large concatameric DNA molecules [56]. In Fig 11, we present an RCA analysis of DNA isolated from PFSK-1: MCVSyn cultures (the same cultures as shown in Figs 8A–8C, 9 and 10B) at 4 or 136 days post transfection (lanes 3–4 and 9–10, respectively). Genomic DNA from the MCC-derived cell lines WaGa and MKL-1 (lanes 5–6 and 7–8, respectively) served as a control for cells harboring integrated viral genomes. Indeed, while no RCA products were observed in mock-transfected PFSK-1 cells or the two MCCL cultures, the material from early and late PFSK-1:MCVSyn cultures yielded efficiently amplified viral DNA.

Collectively, the data presented in Figs 8–11 thus suggest that MCPyV genomes are able to persist as extrachromosomal episomes for several months after transfection into PFSK-1 cells, and furthermore that a miRNA knockout mutant is considerably impaired in its ability to establish long term episomal maintenance.

## Discussion

In this study, we report an in-depth analysis of the MCPyV-encoded miRNA miR-M1 and its functions during short and long-term replication of intact viral episomes. Besides of confirming prior studies which had suggested that mcv-miR-M1 autoregulates T-Ag expression, we demonstrate that mcv-miR-M1 can be expressed independently of NCCR-initiated transcription and uncover an unexpected role for the viral miRNA in episomal persistence.

Our small RNA sequencing data suggest that replicating MCPyV genomes express the viral miRNA to very high levels. In our analysis of MCVSyn-transfected PFSK-1 bulk cultures, mature mcv-miR-M1 species ranked among the top 15 of all miRNAs. Owing to generally low transfection efficiencies achieved with re-circularized genomes, only ~2–5% of cells in such cultures carry the viral genome. Hence, it is likely that mcv-miR-M1 species dominate the spectrum of expressed miRNAs in MCVSyn-positive cells. In contrast, we find that mcv-miR-M1 is expressed at only very low levels in MCC-derived cell lines. The observed frequencies of viral miRNA reads (~0.001%) are in very good accord with those calculated from a recent metastudy of mcv-miR-M1 expression in primary tumor material (0.002%) [19]. Overall, when taking into account transfection efficiencies, mcv-miR-M1 expression levels are estimated to be more than four orders of magnitude higher in cells harboring replicating episomes. Our cross-comparison of mcv-miR-M1 expression levels in PFSK1:MCVSyn and MCCL thus strongly supports the previous notion that the viral miRNA is unlikely to contribute to the progression of MCC via the continuous downregulation of cellular target transcripts [19].

We also find no evidence for the hypothesis that MCC cells may preferentially express an isomiR variant that could target host immune response genes [34]. Our side-by-side comparison of two different library preparation methods rather shows that the relative distribution of miRNA seeds is very similar between MCCL and PFSK1:MCVSyn cells. While the isomiR identified by Lee et al. was indeed the most prominent variant when using one of the two investigated library preparation methods, absolute and relative read counts (Table 1 and S3 Dataset) strongly suggest that the seeming dominance of this isomiR was due to failure to capture the 5p_17-23_ species. The observed discrepancies likely resulted from biases during small RNA library preparation, most notably the influence of small RNA and adapter sequence combinations on the efficiency of 3’ adapter ligation (27–31). Given the difficulties in determining the accurate seed sequences of viral miRNAs, our results thus once more underline the notion that identification of potential host targetomes must be based on unbiased experimental screens with authentic precursors rather than computational prediction alone.

Non-withstanding above considerations, our data show that mcv-miR-M1 expression negatively regulates expression of early messages transcribed from the opposite strand. Similar to SV40, murine PyV and BKPyV [15, 16, 20], a miRNA knockout mutant exhibited appreciably higher levels of LT-Ag expression and DNA replication, but did not produce significantly altered levels of viral progeny (S6 Fig). However, given that the currently available MCPyV replication system generally produces only very low titers of infectious progeny, it remains possible that mcv-miR-M1 may behave differently in a fully permissive system.

Comparison of relative transcript abundance between MCVSyn or MCVSyn-hpko transfected cells suggests that the majority of early transcripts are negatively regulated by miR-M1, as they become more abundant in cells harboring the miRNA knockout (Fig 7). A notable exception is transcript T’5, which is approximately 30fold more abundant in MCVSyn-transfected cells. This observation is consistent with the fact that the d420-a2778 splice removes the sequences complementary to mcv-miR-M1. As the miRNA knockout is expected to selectively destabilize those transcripts which contain target sites, it is to be expected that T’5 accounts for a lower relative fraction of early viral transcripts in MCVSyn-hpko cells. It is also interesting to note that, among the remaining transcripts, those that encode LT-Ag and 57K-Ag (T1 and T4, respectively) appear to be most strongly upregulated in PFSK-1:MCVSyn-hpko cells. This may suggest that they are more efficiently targeted by mcv-miR-M1, e.g. due to secondary structures that facilitate binding of mature miRNAs to their target sites. Certainly, however, further investigation will be required to establish whether this is indeed the case.

Our RNA-seq analysis also identified early strand splice events which originate outside of the major transcriptional cassette defined by 5’- and 3’-RACE analyses. These products are of interest as they may produce dedicated ALTO-encoding messages. While we have formally confirmed the existence of these junctions, RNA-seq coverage also indicates that they are of very low abundance. It is thus unlikely that such transcripts significantly contribute to ALTO production in our system, considering that the protein can be efficiently produced from canonical early region transcripts via leaky scanning [10]. However, it remains possible that increased usage of alternative upstream initiation sites (e.g. that observed at position 115) could elevate transcript abundance. Previous studies from other polyomaviruses such as mouse PyV, SV40 and JCPyV indicate that upstream initiation sites are increasingly used during late stages of the infection cycle [57–59]. Once a fully permissive MCPyV replication system becomes available, it thus will be interesting to study whether similar mechanisms could lead to increased expression of dedicated ALTO-messages during late stages of a productive infection.

We detected two splice junctions that map within the major late strand transcription cassette. While the resulting transcripts are predicted to produce VP1 and VP2, we do not observe splice events which remove the VP2 start codon to produce a dedicated transcript for the putative minor capsid protein VP3. As the VP2 start codon is in a strong Kozak context [9], translation of VP3 from above transcripts via leaky scanning is predicted to be inefficient. Our data are thus in support of a recent study that has concluded MCPyV is unlikely to express a functional VP3 [9].

Interestingly, we find that MCPyV also expresses late strand transcripts with multiple copies of 5’-structures that are reminiscent of the leader-to-leader splice events observed in mouse polyomavirus [50–55]. Similar to the early stages of mouse PyV infection [60], we find that the major transcriptional start site is located downstream of the leader-to-leader splice acceptor. As productive mouse PyV infection proceeds, transcriptional initiation occurs with increasing frequency at alternatives sites which are located upstream of the leader-to-leader splice acceptor [60]. As for early messages, it is thus possible that a similar shift in transcriptional initiation site usage may occur in a fully permissive infection system. Production of leader-to-leader transcripts from primary transcripts which ignore late strand polyadenylation sites has been shown to contribute to the accumulation of late strand transcripts during productive mouse polyomavirus infection [51, 53, 55]. It was suggested that the presence of nuclear antisense RNAs produced from the intron of the late leader-to-leader splice leads to abundant A-to-I editing and subsequent destabilization of early transcripts. Since the processed late mRNA does not contain the complementary intron sequences, it is not subject to editing and thus the ratio of late versus early messages increases [54]. To investigate whether similar mechanisms may occur in our system we scrutinized our RNA-seq data for evidence of A-to-I editing events. Even when allowing 20 mismatches during the alignment step, the rate of A-to-I transitions was below 0.1%. Hence, at least under the conditions and in the semi-permissive system used here, negative regulation of early genes by late strand transcription products appears to proceed predominantly via expression of the viral miRNA. Notably, this does not rule out a role for leader-to-leader splicing, as these events would allow processing of the miRNA from intronic sequences while still preserving the integrity of late strand coding mRNAs. Given that mouse PyV encodes a miRNA at a similar genomic location [15] it appears possible that intron-derived miRNAs could also contribute to accumulation of late mouse PyV mRNAs.

In addition to miRNA expression from canonical late strand transcripts, we also provide evidence for NCCR-independent expression of mcv-miR-M1. Evidence for this conclusion includes (i) identification of a transcriptional initiation site (TI-L2) upstream of the genomic miRNA locus which (ii) exhibits profound enrichment of the histone modification H3K4me3, (iii) autonomous expression of the viral miRNA from subgenomic fragments containing the early coding region and (iv) an approximately 2fold reduction in miRNA expression concomitant with (v) elimination of H3K4me3 peaks upon the introduction of triplet mutations upstream of the genomic mcv-miR-M1 locus. At first, the 2fold reduction in miRNA expression by MCVSyn-pmt may appear unexpected when considering the relatively low frequency of 5’-RACE products observed at TI-L2 (approx. 3%). However, as exonic miRNA processing destroys the precursor transcript, the 5’-RACE protocol can only capture those transcripts which have escaped processing by Drosha. The primary rate of transcriptional initiation events at this site may thus be higher. It should also be noted that, while our data suggest that transcription occurs via RNA polymerase II, we were unable to drive expression of luciferase via the early strand coding fragments upstream of the viral miRNA locus. Hence, it is possible that sequences downstream of the initiation site may be required for efficient expression, perhaps similar to the as of yet undefined genomic pre-miRNA sequence features that can mediate autonomous transcription of some human miRNA loci [61]. The fact that distally initiated transcription through the mcv-miR-M1 locus seems (Fig 5) seems to increase intrinsic promoter activity is also of interest, as such a mechanism could potentially provide a negative feedback for early gene expression. Interestingly, expression of a viral miRNA from an internal promoter has been previously reported for bandicoot papillomatosis carcinomatosis virus type 1 and 2 (BPCV1 and -2, respectively), two viruses which shares distinct features of both the polyomavirus and papillomavirus families [62–64]. In BPCV1 and -2, the non-coding region 2 (NCR2) contains the genomic template as well as promoter for miRNA expression [64]. It was noted that the NCR2 region also contains a predicted LT-Ag consensus binding site (GRGGC), yet whether BPCV LT-Ag indeed binds to this site remains unknown [62–64]. Indeed, the region flanking initiation site TI-L2 contains a cluster of 6 GRGGC pentamers, including two overlapping sites in a head-to-head orientation just 6 nucleotides upstream of the transcriptional start position (S8 Fig). All sites are furthermore perfectly conserved in Gorilla gorilla gorilla polyomavirus 1 (GggPyV1) and Pan troglodytes verus polyomavirus 2 (PtvPyV2a), two close relatives of MCPyV that have been shown to encode orthologues of mcv-miR-M1 [64, 65]. While it is intriguing that, aside from the viral origin of replication, no other locus in the MCPyV, GggPyV1 or PtvPyV2a genomes exhibits a similar accumulation of GRGGC pentamers, we clearly did not observe LT-Ag peaks upstream of the viral miRNA in our ChIP-seq experiments (Fig 6). Hence, if LT-Ag indeed binds to these sites under the conditions used here, it must do so transiently or with significantly reduced affinity compared to the viral origin of replication. Considering all of the above, additional experiments will undoubtedly be required to fully characterize factors and features which regulate NCCR-independent expression of mcv-miR-M1.

If mcv-miR-M1 expression can occur independent of late gene expression, then why do integrated genomes in MCC fail to efficiently express the viral miRNA? While we presently can only speculate, given that high level early antigen expression is required for survival of MCC cells [66] we would predict that tumor progression selects for silencing of the viral miRNA promoter. We are currently evaluating the chromatin status of integrated genomes to investigate this scenario.

Perhaps the most intriguing finding of our study is that, in the absence of any selection pressure, wild type MCPyV genomes can persist in continuously growing cultures for more than 6 months after the initial transfection. In contrast, the mcv-miR-M1 knockout mutant was consistently lost at an accelerated rate. The fact that MCVSyn genome copy numbers reached a stable plateau phase in only one out of the three independently performed experiments, however, also suggests that long-term maintenance is affected by as of yet unknown stochastic events. While the results presented in Fig 11 clearly argue against chromosomal integration constituting such an event, another possibility would be the accumulation of adaptive mutations or genomic rearrangements in long term cultures. The fact that long term maintenance did not require selection would seem to argue against such a possibility. More importantly, however, we have subjected RCA amplification products from long-term cultures to high-throughput sequencing and found their sequences to be 100% identical to those of the input genomes. It is interesting to note that the observations made here bear some resemblance to the latency establishment phase of the gammaherpesviruses EBV and KSHV. While incoming genomes rapidly adopt latent gene expression profiles, EBV as well as KSHV episomes exhibit an accelerated loss rate during the first few weeks of infection until rare epigenetic events of hitherto unknown nature lead to stabilization of episomes and subsequent long term maintenance [67–69]. Considering the transient increase in copy numbers observed at day 84 of the experiment shown in Fig 8A–8C, it is tempting to speculate that a stochastic event occurring at this time point may have allowed subsequent stabilization of MCPyV episomes. Unfortunately, due to the very low frequency of MCPyV-positive cells at this or later time points we were unable to investigate the chromatin state of stable viral episomes in these cultures. We are currently striving to enrich cells harboring stable MCPyV episomes to allow a detailed investigation of their epigenetic landscape and gene expression profile. It should be pointed out that we currently cannot exclude the possibility that MCPyV maintenance may also involve continuous shedding of infectious virions. Thus, long term maintenance may result from latency-like episomal persistence, continuous low level infection of initially untransfected cells (or cells having lost the viral genome), or a combination of such processes. However, in our view the fact that PFSK-1 cells do not allow efficient particle production and serial transmission together with the observation that mcv-miR-M1 deficiency does not affect the levels of viral progeny (S6 Fig) argues against continuous virion shedding as the primary maintenance mechanism. We are currently performing long term studies with additional MCPyV mutants to investigate whether persistence requires virion production.

Why is the miRNA-knockout mutant MCVSyn-hpko impaired in long-term persistence? Several possibilities come to mind. Firstly, given that small RNAs can induce local chromatin changes via hitherto poorly understood mechanisms [70–73], viral miRNA expression may negatively affect a potentially stabilizing event as discussed above. While formally possible, we do not consider this possibility to be very likely. We instead favor a second and much simpler possibility: That continued mcv-miR-M1 expression prevents accumulation of LT-Ag protein to levels which impair cell growth. It has previously been shown that LT-Ag can have growth promoting activities, but via carboxyterminal sequences may also inhibit proliferation if expressed at high levels [74]. We have formally confirmed that PFSK-1 cells respond to LT-Ag expression with a dose dependent decrease in proliferation rates (S9 Fig). Hence, we suspect that lack of the viral miRNA results in aberrantly high LT-Ag expression, leading to accelerated loss of MCPyV-positive cells and a decreased probability that genome stabilization may occur. This model provides a convenient explanation for the fact that MCVSyn-hpko genomes exhibit increased DNA replication at early time points, but nevertheless exhibit accelerated loss in long-term cultures. Lastly, a third (and not mutually exclusive) possibility is that, similar to some herpesvirus miRNAs [22, 23, 75–77], mcv-miR-M1 may downregulate host transcripts to create a cellular environment that is supportive of long-term episomal maintenance. Although attractive, experimental identification of candidate host targets (preferably via unbiased screens) will be required to substantiate such a scenario.

What is the biological significance of the observed ability of MCPyV episomes to persist over extended periods of time? It is tantalizing to speculate that MCPyV may have evolved similar mechanisms as papillomaviruses to persist in a non-vegetative state of infection. However, it must be pointed out that it is currently unclear to what extend the cell system used here adequately reflects the behavior of MCPyV-infected cells *in vivo*. While PFSK-1 cells (like all other cell lines or-types tested thus far) do not support lytic growth of MCPyV, the precise cells types in which the virus establishes productive and/or persistent infections *in vivo* remain unknown. Since, obviously, long term persistence would not provide a significant benefit in fully permissive infected cells (which would likely die within a few days after initial infection), one would thus have to postulate that there may be semi-permissive host cell types (or differentiation stages) in which the virus establishes latent or quasi-latent infections. Although we find this to be a very intriguing possibility, verification of such models will ultimately have to await identification of the authentic *in vivo* target cells in healthy carriers.

Finally, are the findings reported here of any relevance for the pathogenesis of MCC? At present, the limited amount of available data does not allow us to draw such a conclusion. Within the limits of the caveats discussed above, we propose that, similar to BKPyV [20], the physiological function of the MCPyV miRNA may be to augment viral persistence which, given the existence of an autonomous promoter, may proceed in a non-vegetative state of infection. If so, one may also speculate that prolonged episomal persistence could increase the overall chance of integration events that are expected to be extremely rare during natural infection, but which are a hallmark of all MCPyV-positive MCC. Thus, while spurious expression of the viral miRNA is likely to be inconsequential once integration has occurred, in the above scenario mcv-miR-M1 may very well make an indirect contribution by supporting long-term persistence of viral episomes. Of note, LT-Ag has been demonstrated to directly interact with the bromodomain protein BRD4, a factor which is targeted by several papillomavirus E2 to mediate episomal tethering during persistent infection [78–83]. Although highly speculative, given recent findings suggesting a role for the E2/BRD4 interaction during papillomavirus integration [84], one may envision that the interaction between BRD4 and LT-Ag could also more directly contribute to MCPyV integration events. Indeed, our own preliminary studies indicate that LT-Ag binds to a large number of host chromosome loci in a non-random manner. We are currently investigating whether such loci may also represent preferred sites of chromosomal integration in MCC. In the meantime, the findings reported here open new lines of investigation that are expected to significantly improve our understanding of the MCPyV lifecycle.

## Methods

### Cell lines and tissue culture

PFSK-1 cells (ATCC, CRL-2060) and MCC cell lines (MKL-1 [85] and WaGa [8]) were maintained in RPMI medium supplemented with 10% fetal calf serum (FCS) and 5% penicillin/streptomycin. HEK293 cells [86] were cultured in Dulbecco’s modified Eagle’s medium (DMEM) supplemented with 10% FCS and 5% penicillin/streptomycin. All cells were grown in a 5% CO_2_ humidified atmosphere at 37°C.

### Plasmids and oligonucleotides

Construction of the synthetic consensus clone pMK-MCVSyn used for production of re-circularized MCPyV genomes has been described previously [27]. To generate the hairpin mutant MCVSyn-hpko, a 384 bp fragment spanning nucleotides 1118–1501 of the MCVSyn genome and containing 14 nucleotide substitutions in the region encoding mcv-miR-M1 (S1 Fig) was synthetically generated (GeneART, Regensburg). A fragment spanning the same genomic region, but containing 68 nucleotide exchanges (S1 Fig) in the suspected promoter region upstream of the mcv-miR-M1 locus was synthesized to generate the mutant genome MCVSyn-pmt. Both fragments were inserted into pMK-MCVSyn using *BamHI* and *SanDI* restriction sites.

Plasmid pER was produced by amplification of the MCPyV early coding region from MCVSyn using primers MCPyV EcoRV F/MCPyV XhoI R and subsequent cloning into the pCR2.1 plasmid (life technologies). To generate plasmid pER-pmt, the region upstream of the viral miRNA locus in pER was substituted by the mutated region from MCVSyn-pmt. Plasmids pCMV:ER-AS and pCMV:ER-S were generated by excision of the MCPyV early coding region from plasmid pER and directional cloning into pCDNA3.1.

All primer- and oligonucleotide-sequences used in this study are listed in S1 Table.

### Re-circularization and transfection of MCPyV genomes

Re-circularization and transfection were carried out as described previously [27]. Briefly, the bacterial backbone of pMK-MCVSyn, pMK-MCVSyn-hpko or pMK-MCVSyn-pmt plasmids was excised by *SacI* digestion (FastDigest, Thermo Scientific). After gel purification of the linearized viral genomes, intramolecular ligation was performed using T4 DNA Ligase (Thermo Scientific), followed by spin column purification of the recircularized DNA. 3x10^5^ PFSK-1 cells were seeded in 6-well dishes one day prior to transfection. 200 ng of re-circularized viral DNA together with 500 ng carrier DNA (pUC18) were transfected using X-tremeGene HP DNA Transfection Reagent (Roche) following the manufacturer’s instructions. Mock treated cells were transfected with equivalent amounts of carrier DNA only. Cells were harvested for analysis at the indicated time points.

### MCVSyn replication assays and copy number quantification

*DpnI* resistance/replication assays were performed after HIRT extraction of low molecular weight DNA (HIRT DNA) as previously described [87]. Extracted DNA was subsequently digested with *EcoRI* and *DpnI* (FastDigest, Thermo Scientific) for 60 min at 37°C. 1 μg of the resulting DNA was separated on a 0.8% agarose gel and transferred to a nylon membrane (Zeta-Probe GT Membrane, Bio-Rad). For detection of MCVSyn DNA, a genomic fragment was amplified from the early viral region using primers MCPyVLT fw and MCPyV LT rev and labeled with ^32^P dCTP (Rediprime II DNA Labeling System, GE Healthcare). Blots were hybridized with the labeled probe for 16h at 42°C in ULTRAhyb buffer (Ambion). Blots were washed 2x 20 min with 1% SSC, 0.1% SDS and 2x20 min with 0.1x SSC, 0.1% SDS at 50°C. After at least 24 h of exposure, blots were scanned with the Fuji phosphorimager FLA7000 and analyzed with Multigauge software.

For the determination of MCPyV genome copy numbers, genomic DNA (gDNA) was extracted from isolated nuclei of transfected cells. Nuclei were prepared by adding 2x lysis buffer (0.65 M Sucrose, 20 mM Tris-HCl pH 7.8, 10 mM MgCl_2_, 2% Triton-X 100) to a final concentration of 1x to trypsinized cells resuspended in PBS. After 5 min incubation on ice, nuclei were centrifuged and resuspended in 50 μL PBS. 300 μL gDNA lysis buffer (100 mM NaCl, 10 mM Tris-HCl pH 8, 25 mM EDTA pH 8, 0.5% SDS) supplemented with 200 μg proteinase K (Peqlab) were added, followed by incubation at 54°C for 16 h. The DNA was purified by phenol/chloroform extraction and isopropanol precipitation. After treatment with RNase A (Peqlab) for 30 min at 37°C gDNA was digested with *EcoRI* and *DpnI* (FastDigest, Thermo Scientific) for 60 min at 37°C.

25 ng gDNA were used as input for quantitative PCR. Viral genomes were quantified with primers binding to the late region (MCPyV VP1 fw and MCPyV VP1 rev), spanning three *DpnI* restriction sites. *SacI* digested, linear MCVSyn DNA was used to generate a standard curve with a defined number of viral genomes. The number of viral genomes was normalized to GAPDH locus copy numbers (primers: GAPDH DNA fw and GAPDH rev), which were determined using a standard curve with a defined number of GAPDH locus copies.

### Rolling circle amplification (RCA)

Rolling circle amplification was performed with the TempliPhi 100 amplification kit (Amersham Biosciences) according to the manufacturer's instructions with additional 450 μM dNTP as described in Rector et al. 2004 [88]. RCA products were digested with restriction enzymes, separated on a 0.8% agarose gel and analyzed by ethidium bromide staining. Libraries of RCA products for HTS analysis were generated with the NEBNext Ultra DNA Library Prep Kit for Illumina and sequenced on an Illumina HiSeq2500 instrument. Reads were subjected to de novo assembly using the Trinity package (v r2013-02-25) [89] and resulting full-length MCPyV genomes (average coverage >200) were compared to input sequences by blast analysis (BLAST Plus Package v 2.2.28).

### Small RNA northern blot analysis and quantitative stem-loop PCR

For detection of small RNAs by northern blotting, total RNA was harvested using RNABee (AMS Biotechnology) according to the manufacturer's instructions. 14 μg of total RNA were separated on a denaturing 15% polyacrylamide urea gel and transferred to Zeta-Probe GT membranes (Bio-Rad) by electro blotting. Blots were hybridized to a ^32^P dATP labeled antisense oligonucleotide probe (mcv-miR-M1 probe, S1 Table) in ExpressHyb (BD Biosciences Clontech) hybridization buffer for 16h at 37°C. Membranes were washed twice in 2x SSC, 0.1% SDS at room temperature and subsequently subjected to autoradiography. Blots were scanned on the BAS-Reader and analyzed with AIDA Software.

For quantitative stem-loop RT-PCR, reverse transcription (RT) was performed as described by Varkonyi-Gasic et al. [90] using 1 μg of total RNA as input for each sample and a mcv-miR-M1 specific stem-loop primer (SL mcv-miR-M1). For normalization, a reverse primer for GAPDH (GAPDH rev) was included in the RT reaction. 1.5 μL cDNA per sample were analyzed by real-time PCR on the Rotor-Gene Q (Qiagen) using the Rotor-Gene Multiplex PCR Mastermix (Qiagen) and the following primer pairs: mcv-miR-M1: mcv-miR-M1 fw/universal rev; GAPDH: GAPDH BSP fw/GAPDH rev. Differently labeled TaqMan probes (Taqman probe mcv-miR-M1, Taqman probe GAPDH) were used to quantify the expression of mcv-miR-M1 and GAPDH in the same real-time PCR reaction. Analysis of real-time PCR experiments was carried out with Rotor-Gene Q software (Qiagen).

### Chromatin immunoprecipitation (ChIP) assays

Chromatin Immunoprecipitation (ChIP) was performed as previously described [91, 92]. In brief, 4d post transfection with MCVSyn wt or MCVSyn mutants, chromatin of 1×10^6^ cells was crosslinked by incubation with 1% formaldehyde. The reaction was stopped by the addition of glycine. Chromatin was extracted from isolated nuclei and fragmented by sonication (Bioruptor, Diagenode) to an average length of 200–500 bp. A fraction of the total chromatin sample was set aside for the preparation of input control. The remaining material was pre-cleared with BSA blocked protein-G sepharose beads (GE Healthcare) to reduce non-specific background binding. For immunoprecipitation, 2 μg of antibodies specific for the histone modification H3K4-me3 (Millipore, #04–745) or for MCPyV LT-Ag (CM2B4, Santa Cruz Biotechnology, sc-136172) or IgG anti-rabbit (Millipore, #12–370) antibody were added to the chromatin and incubated for 16 h at 4°C. Chromatin-immunocomplexes were precipitated by the addition of protein-G sepharose beads, washed with increasing salt concentrations, eluted and de-crosslinked for 16 h at 65°C. DNA was purified by phenol-chloroform extraction and ethanol precipitation. For HTS analysis, libraries were prepared from ChIP samples using the NextFlex ChIP-Seq kit (Bioo Scientific) and sequenced on the Illumina HiSeq2500. Reads were mapped to MCVSyn or MCVSyn-pmt genome sequences using Bowtie (v 0.12.9).

### Western blot and immunofluorescence analysis

For Western Blotting, MCVSyn transfected cells were harvested at the indicated time points and resuspended in lysis buffer (50 mM Tris pH 8.0, 150 mM NaCl, 1% NP40, 0.5% Na-Deoxycholat, 5 mM EDTA, 0.1% SDS, proteinase inhibitor cocktail, Roche). 25 μg of protein were separated by SDS-PAGE (10% gels) and electroblotted on a PVDF-membrane. Blots were incubated with MCPyV LT-Ag antibody CM2B4 (Santa Cruz, sc-136172).

For immunofluorescence analyses, PFSK-1 cells grown on coverslips were fixed with methanol at room temperature for 30 min and rinsed in PBS for 10 min. Fixed cells were blocked with 4% BSA in PBS for 30 min and then incubated with a 1:50 dilution of the LT-Ag antibody CM2B4 in 4% BSA in PBS/0.05% Tween-20 for 2 h. Coverslips were washed three times in PBS for 10 min each, followed by incubation with a 1:1000 dilution of goat anti-mouse Alexa Fluor 555-conjugated secondary antibody (Life Technologies, A 21422) in 4% BSA in PBS/0.05% Tween-20 for 2 h. Coverslips were washed three times in PBS for 10 min each, counterstained and mounted with vectashield mounting medium with DAPI (Vector, H-1200). Images were acquired with a confocal laser-scanning microscope (Nikon C2+).

### FISH analysis

5x10^4^ cells were cytospun for 5 min at 900 rpm on Superfrost/Plus slides (Fisher) and fixed in methanol, followed by digestion (0.01% pepsin, 0.01 N HCl) for 5 min at 37°C and RNase A incubation (100 μg/ml) in 2x SSC for 1 h at 37°C. After washing in PBS and refixation (3% formaldehyde/PBS, 50mM MgCl_2_), slides were passed through a dehydration series of 70%, 85%, and 100% ethanol for 5 min each and air dried. For denaturation, slides were incubated in 70% formamide in 2x SSC for 5 min at 73°C and then promptly placed in ice-cold 70% ethanol for 5 min, and dehydrated again as described above.

1 μg of MCVSyn DNA was labeled with Dig-Nick Translation Mix (Roche, 11745816910) according to the manufacturer’s instructions. Labeled DNA was ethanol precipitated in the presence of excess sonicated salmon sperm DNA (Life Technologies). The final product was resuspended in hybridization buffer (50% formamide and 10% dextran sulfate in 2x SSC) to a final concentration of 10 ng/μl and stored at -20°C. 5 μl (50ng) of the probe were heat-denatured for 5 min at 73°C and placed under a coverslip on the appropriate area of the slide. The coverslip was fixed with fixogum (Marabu, 290110000). Slides were hybridized in a humid chamber overnight at 37°C.

After hybridization, slides were washed three times (2x SSC, 0.2% Tween) for 2 min each, twice at 20°C and in between at 70°C, followed by blocking with 4% BSA/PBS for 30 min at 37°C and incubation with sheep-anti-Digoxigenin-FITC-antibody (Roche, 11207741910), diluted 1:50 in 4% BSA/PBS with 0.2% Tween, for 2hr at 37°C in the dark. Slides were washed with PBS/0.2% Tween three times for 10 min each at 20°C in the dark, counterstained and mounted with vectashield mounting Medium with DAPI (Vector, H-1200). Images were acquired with a confocal laser-scanning microscope (Nikon C2+).

### RACE analysis

3’-RACE analysis was performed according to the protocol of Scotto-Lavino et al. [93] with minor modifications. In brief, 5 μg of total RNA of MCVSyn transfected PFSK-1 cells 4d post transfection were subjected to cDNA synthesis using Superscript III (Invitrogen) and an anchored oligo-dT primer (Q_T_). The input RNA was digested by addition of 1.5 U RNAse H (NEB) and incubation at 37°C for 20 min. For the first round of amplification, a gene specific primer (LT_o_/VP1_o_) and a primer specific for the sequence of the Q_T_ primer were used. A second round of amplification with nested gene specific reverse primers (LT_i_/VP1_i_) and the forward primer Q_i_ was used to increase specificity and add restriction sites to the ends of the PCR product. After digestion with the respective restriction enzymes (Fast Digest, Thermo scientific), RACE products were cloned into pCR2.1 plasmid. After transfection into bacteria, individual clones were subjected to Sanger sequencing.

5’ RACE analysis of MCVSyn transfected PFSK-1 cells was performed with the GeneRacer Kit protocol (Invitrogen) according to the manufacturer’s instructions. Briefly, 5 μg of total RNA were dephosphorylated, decapped and then ligated to a 5’ RACE RNA adapter. cDNA was synthesized with gene specific primers (early/late region rev, S1 Table) using Superscript III according to the manufacturer’s instructions. After touchdown PCR with gene specific primers, nested PCR was performed by which restriction sites were added at both ends of the amplification products (primers: early/late region rev nested). All RACE PCR amplifications were performed with Pfu Ultra II (Agilent technologies) according to the manufacturer’s protocol. 5’ RACE products were analyzed by HTS after library preparation with the NEBNext Ultra DNA Library prep Kit for Illumina. Reads were mapped to the MCVSyn genome with TopHat2 [94].

### Small RNA sequencing

For sequencing of small RNA moieties, RNA from MCVSyn transfected cells and MCC cell lines was subjected to library preparation using the TruSeq Small RNA Sample Preparation Kit (Illumina) or the NEBNext Small RNA Library Prep Set for Illumina. Small RNA libraries were sequenced on the Illumina HiSeq platform. After adapter trimming, mapping of reads to the MCVSyn genome and quantification of mature miRNA deposited in the miRNA registry (miRBase) release 21 [95] were performed using CLC Genomics Workbench v7.5.1 (Quiagen), allowing an offset of 5 nucleotides of mature miRNAs along the precursor to ensure detection of isomiRs.

### RNA sequencing (RNA-seq)

Library preparation for strand specific RNA sequencing was carried out using the NEXTflex Directional RNA-Seq Kit (Bioo Scientific) according to the manufacturer’s instructions. Libraries were sequenced on the Illumina HiSeq 2500 platform. To allow detection of splice events that extend over the origin, reads were mapped to two concatenated copies of the MCVSyn or MCVSyn-hpko genomes using TopHat2 v 2.0.13 [94]. The positions of mapped reads and junctions were subsequently collapsed back on unit-length genomes. From the resulting SAM files, we counted the number of unspliced reads that extended over splice sites of junctions detected by TopHat to determine splice site efficacy and frequency of individual junctions. To estimate transcript abundance, for each combination of splice junctions that mapped within either the major early or late transcription cassettes we calculated a relative strand-specific combinatorial frequency value by multiplying observed frequency values for individual donor sites. The relative ratio of late to early transcripts was subsequently estimated by calculation of normalized RPKM (reads per kilobase per million mapped reads) for each of the transcripts.

### Preparation of DNaseI resistant DNA from cell culture supernatants

Four days after transfection with re-ligated MCVSyn, cell culture supernatants were collected and sterile filtered. 1 ml of supernatant was supplemented with 10x DNaseI reaction buffer and DNA was digested with 10 μl DNase I (amplification grade, Invitrogen) for 1 h at 25°C. DNase I was heat inactivated for 10 min at 65°C in the presence of 2.5 mM EDTA. Proteins were degraded by addition of 5 μl Proteinase K and 1% SDS at 50°C for 16h. DNA was retrieved by phenol-chloroform extraction and precipitation.

### Re-infection assays

For re-infection assays, PFSK-1 cells were transfected with MCVSyn or MCVSyn-hpko. 8 days post transfection; cells were lysed by three freeze-thaw-cycles. Cell debris was removed by centrifugation and lysates were passed through a 0.22 μm filter. Lysates prepared from a 10 cm dish were used to inoculate one 6-well of freshly seeded PFSK-1 cells. 24h post infection, medium was changed and cells were incubated for additional 3–7 days as indicated prior to DNA isolation.

### Proliferation assays

PFSK-1 cells in 10 cm dishes were transfected with the indicated amounts of a pCDNA3.1 LT-antigen expression plasmid added up to 10 μg of total plasmid DNA with an empty pCDNA3.1 plasmid.

At 24h post transfection, cells were seeded in 96-well plates and grown for another 24h. For measurement of proliferation, cells were incubated with 10 μl MTT reagent (Chemicon) per well for 4h. Afterwards, cell culture medium was removed and the formazan crystals were resuspended in 200 μL DMSO. Absorbance was measured at 540 nm with a reference wavelength of 690 nm.

### Accession numbers

All MCPyV sequences and genome coordinates in this study refer to the MCVSyn genome, which is 100% identical to the prototypical MCPyV field strain R17b (genbank accession numbers JN707599 and NC_010277, respectively). The accession numbers for Gorilla gorilla polyomavirus 1 (GggPyV1) and Pan troglodytes verus polyomavirus 2 (PtvPyV2a) sequences as shown in S8 Fig are HQ385752.1 and HQ385748.1, respectively. Primary read and mapping data of all small RNA-seq, RACE-seq, ChIP-seq and mRNA-seq experiments performed in this study are publicly available at the European Nucleotide Archive (ENA, http://www.ebi.ac.uk/ena) under accession numbers PRJEB9667 (small RNA-seq data), PRJEB9666 (RACE-seq data), PRJEB9670 (ChIP-seq data) and PRJEB9669 (mRNA-seq data).

## Supporting Information

S1 Fig Mutations introduced in MCVSyn-hpko and MCVSyn-pmt. **(A)** Schematic depiction illustrating the location of mutated regions (symbolized by open boxes at the top) in MCVSyn-hpko and MCVSyn-pmt. **(B)** Alignment of nucleotide sequences and translation products from the LT-Ag and ALTO open reading frames in MCVSyn (wt), MCVSyn-hpko and MCVSyn-pmt. Nucleotide and amino acid substitutions are shown in red. All nucleotide substitutions preserve the LT-Ag coding sequence. Nucleotide substitutions introduced in MCVSyn-hpko result in a total of 10 amino acid substitutions between aa positions 101 and 119 of ALTO. In MCVSyn-pmt, to avoid potential pleiotropic effects due to introduction of a large number of amino acid substitutions in ALTO, the first mutation was designed to create a stop codon in the ALTO ORF. The expected protein product is truncated after aa position 133 and is likely to be non-functional due to the lack of the conserved carboxyterminal region.
(TIF)

S2 Fig Confirmation of novel early and late splice junctions. Agarose gel image of RT-PCR products from RNA of MCVSyn or mock-transfected PFSK-1 cells isolated at 4d post transfection. Fragments containing the following splice junctions were amplified by using exon boundary-spanning primers: *Lanes 1 and 2*: splice junction d5335-a861(+), expected fragment size 231 bp (primers d5335-a861 BSP fw/rev); *Lanes 3 and 4*: splice junction d141-a861(+), expected fragment size 255 bp (primers d141-a861 BSP fw/rev); *Lanes 5 and 6*: splice junction d1142-a5308(-), expected fragment size 269 bp (primers d1142-a5308 fw/BSP rev). *Lanes 7 and 8*: splice junction d5145-a5308(-) (leader-leader-splice), expected fragment size 110 bp (primers d5145-a5308 fw/BSP rev). In addition to unit length amplification products (marked with an asterisk), higher molecular weight products that are likely to contain multiple copies of leader sequences and leader-to-leader splice junctions are visible.
(TIF)

S3 Fig Mutations introduced upstream of the mcv-miR-M1 locus reduce autonomous miRNA-expression. GAPDH-normalized expression of mcv-miR-M1 (as measured by stem-loop RT-qPCR) in PFSK1-cells after 2d of transfection with plasmids pER or pER-pmt (left and center, respectively), or in mock transfected cells (right). Mean values and standard deviations were calculated from six independent experiments, and significance of reduced miRNA expression was evaluated using unpaired t-test.
(TIF)

S4 Fig RNA-seq coverage plots. (**A**) Schematic depiction of the MCPyV genome. **(B, C)** RNA-seq coverage of PFSK-1 cells transfected for four days with either MCVSyn or MCVSyn-hpko (top and bottom graphs in each panel, respectively). A and B represent two independently performed experiments in which PFSK-1 cells were in parallel transfected with either wt or mutated genomes. Read coverage on the early (positive axis; blue) or late strand (negative axis; red) is shown relative to the maximally observed nucleotide coverage (set to 100%) across the viral genome. Note that the negative/late strand axis is shown at a lower scale for results from MCVSyn-hpko transfected cells to facilitate comparison of late strand coverage profiles.
(TIF)

S5 Fig Donor and acceptor consensus sequences at splice junctions. MCPyV donor (**A**) or acceptor (**B**) sites (shown in bold) identified by Shuda et al. (marked with an asterisk) [8] or in this study. Matches to consensus splice site sequences (shown underneath each donor or acceptor site) are indicated by plus signs. Scores shown to the right of each site were calculated using the AST tool (http://ibis.tau.ac.il/ssat/SpliceSiteFrame.htm).
(TIF)

S6 Fig MCVSyn and MCVSyn-hpko produce comparable levels of viral progeny. **(A)** Viral genome copy numbers from total genomic DNA (gDNA) or DNaseI treated supernatants (SN) of PFSK-1 cells at 4d after transfection with MCVSyn or MCVSyn-hpko. Mean values and standard deviations were calculated from three independent experiments. **(B)** Lysates prepared by freeze-thaw lysis from PFSK-1 cells at 8 days post transfection with MCVSyn wt or MCVSyn-hpko were used to inoculate fresh PFSK-1 cultures. MCVSyn copy numbers per cell were determined at 4d and 8d post infection by qPCR and are shown relative to copy numbers in the transfected cultures from which the lysates were derived (input, set to 100%). Mean values and standard deviations were calculated from 3 independently performed experiments.
(TIF)

S7 Fig FISH analysis of MCPyV genome in long term cultures of MCVSyn transfected cells. Shown are exemplary lower magnification FISH images derived from PFSK-1 cultures at the indicated time points after transfection with MCVSyn.
(TIF)

S8 Fig Conservation of GRGGC motifs at the transcriptional initiation site TI-L2. Alignments show genomic sequences upstream of the miRNA loci (shaded grey) in MCPyV and the related polyomaviruses Gorilla gorilla polyomavirus 1 (GggPyV1) and Pan troglodytes verus polyomavirus 2 (PtvPyV2a) [64, 65]. GRGGC pentamers are shown in bold and marked by red block arrows. Additional GGAGC or GAGCC sequences as observed at the imperfect P3 site of the MCPyV origin of replication [41, 42] are marked by blue block arrows. The location of the transcriptional initiation site TI-L2 is marked with a solid arrow.
(TIF)

S9 Fig High-level LT-Ag expression inhibits growth of PFSK-1 cells. PFSK-1 cells were transfected with increasing amounts of a LT antigen expression construct (pCMV:ER-S) and proliferation was measured by MTT-assays at the indicated time points. Proliferation rates were calculated relative to the first measurement at 24h post transfection. The total amount of transfected DNA was brought to 10ug with pUC18 DNA in each transfection (‘mock’ hence indicates transfection of 10ug pUC 18 only).
(TIF)

S1 Table Primers and probes used in this study. (DOCX)

S1 Dataset miRNA-seq coverage data. The dataset provides miRNA-seq read coverage data across the forward and reverse strand of MCVSyn genomes from PFSK-1:MCVSyn, WaGa or MKL-1 cells. Small RNA libraries were prepared with NEBNext or Truseq kits as indicated.
(XLSX)

S2 Dataset miRNA-seq rank data. Ranked mature miRNAs as detected in PFSK-1:MCVSyn, WaGa or MKL-1 when using NEBNext or Truseq library preparation protocols. ‘Score’ values indicate the number of reads mapped to a given miRNA.
(XLSX)

S3 Dataset mcv-miR-M1 seed distribution. Distribution of mature miRNA reads exhibiting the indicated seed sequences (left columns), arranged according to their relative position in the mcv-miR-M1 stem loop precursor encoded by nucleotides 1251 to 1168 of the MCVSyn genome. Read counts are given for each seed as observed in PFSK-1:MCVSyn, WaGa or MKL-1 when using NEBNext or Truseq library preparation protocols.
(XLSX)

S4 Dataset 5’-RACE read coverage. Coverage data of 5’-ends of reads from early and late strand 5’-RACE products mapped across the MCPyV genome.
(XLSX)

S5 Dataset ChIP-seq coverage. LT-Ag, H3K4me3 and IgG ChIP-seq read coverage across the viral genome from MCVSyn or MCVSyn-pmt-transfected PFSK-1 cells.
(XLSX)

S6 Dataset RNA-seq coverage. RNA-seq coverage from MCVSyn or MCVSyn-hpko-transfected PFSK-1 cells along the early and late strands of the viral genome. ‘Experiment I’ and ‘-II’ denote two independently performed side-by-side transfections of the wt or mutant genomes.
(XLSX)
