# Supplementary figures and images for: A Comprehensive Analysis of Replicating Merkel Cell Polyomavirus Genomes Delineates the Viral Transcription Program and Suggests a Role for mcv-miR-M1 in Episomal Persistence
Source: PLoS Pathog. 2015 Jul 28;11(7):e1004974. doi: 10.1371/journal.ppat.1004974 (PMC4517807; doi:10.1371/journal.ppat.1004974)

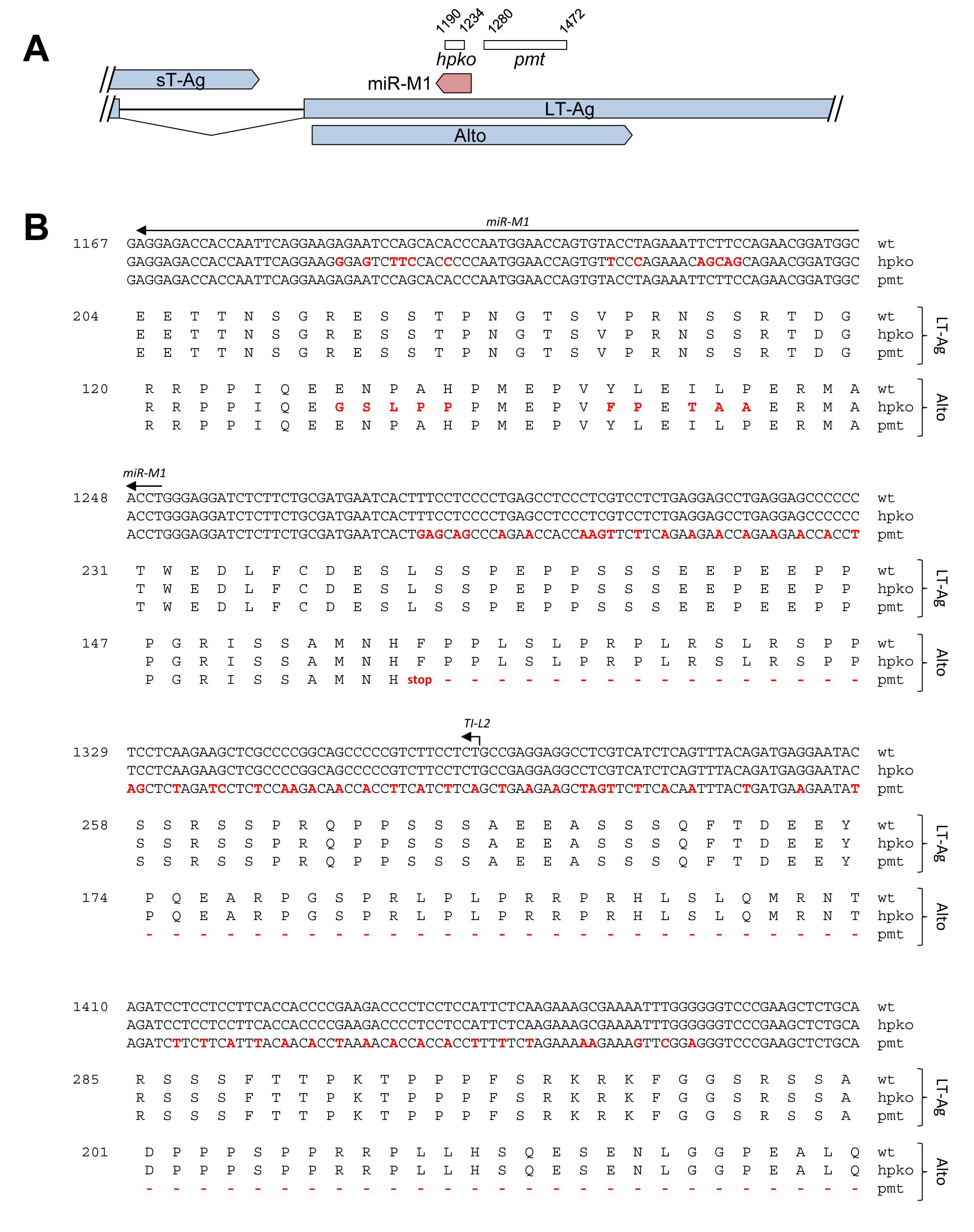

Supplement: S1 Fig — (A) Schematic depiction illustrating the location of mutated regions (symbolized by open boxes at the top) in MCVSyn-hpko and MCVSyn-pmt. (B) Alignment of nucleotide sequences and translation products from the LT-Ag and ALTO open reading frames in MCVSyn (wt), MCVSyn-hpko and MCVSyn-pmt. Nucleotide and amino acid substitutions are shown in red. All nucleotide substitutions preserve the LT-Ag coding sequence. Nucleotide substitutions introduced in MCVSyn-hpko result in a total of 10 amino acid substitutions between aa positions 101 and 119 of ALTO. In MCVSyn-pmt, to avoid potential pleiotropic effects due to introduction of a large number of amino acid substitutions in ALTO, the first mutation was designed to create a stop codon in the ALTO ORF. The expected protein product is truncated after aa position 133 and is likely to be non-functional due to the lack of the conserved carboxyterminal region. (TIF) [file ppat.1004974.s001.tif]

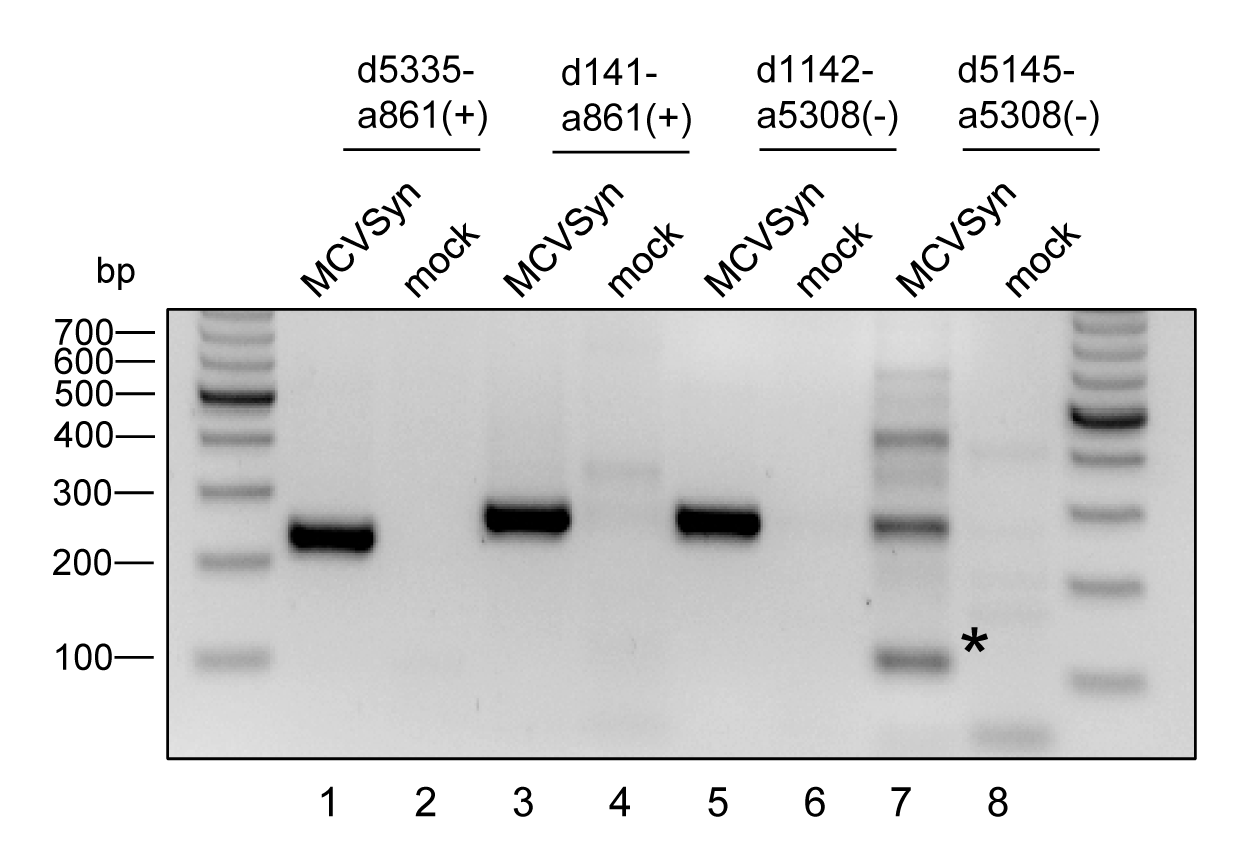

Supplement: S2 Fig — Agarose gel image of RT-PCR products from RNA of MCVSyn or mock-transfected PFSK-1 cells isolated at 4d post transfection. Fragments containing the following splice junctions were amplified by using exon boundary-spanning primers: Lanes 1 and 2: splice junction d5335-a861(+), expected fragment size 231 bp (primers d5335-a861 BSP fw/rev); Lanes 3 and 4: splice junction d141-a861(+), expected fragment size 255 bp (primers d141-a861 BSP fw/rev); Lanes 5 and 6: splice junction d1142-a5308(-), expected fragment size 269 bp (primers d1142-a5308 fw/BSP rev). Lanes 7 and 8: splice junction d5145-a5308(-) (leader-leader-splice), expected fragment size 110 bp (primers d5145-a5308 fw/BSP rev). In addition to unit length amplification products (marked with an asterisk), higher molecular weight products that are likely to contain multiple copies of leader sequences and leader-to-leader splice junctions are visible. (TIF) [file ppat.1004974.s002.tif]

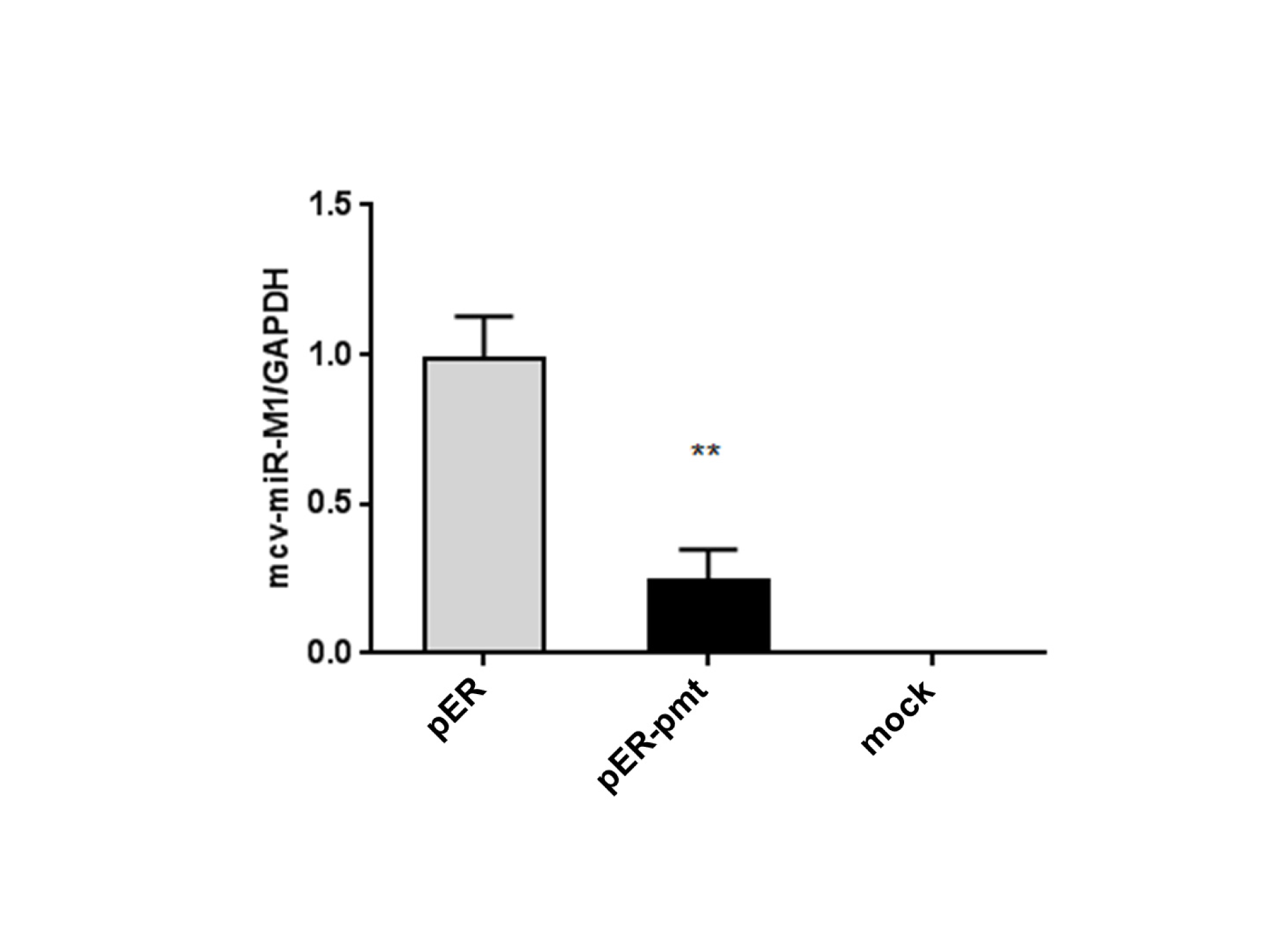

Supplement: S3 Fig — GAPDH-normalized expression of mcv-miR-M1 (as measured by stem-loop RT-qPCR) in PFSK1-cells after 2d of transfection with plasmids pER or pER-pmt (left and center, respectively), or in mock transfected cells (right). Mean values and standard deviations were calculated from six independent experiments, and significance of reduced miRNA expression was evaluated using unpaired t-test. (TIF) [file ppat.1004974.s003.tif]

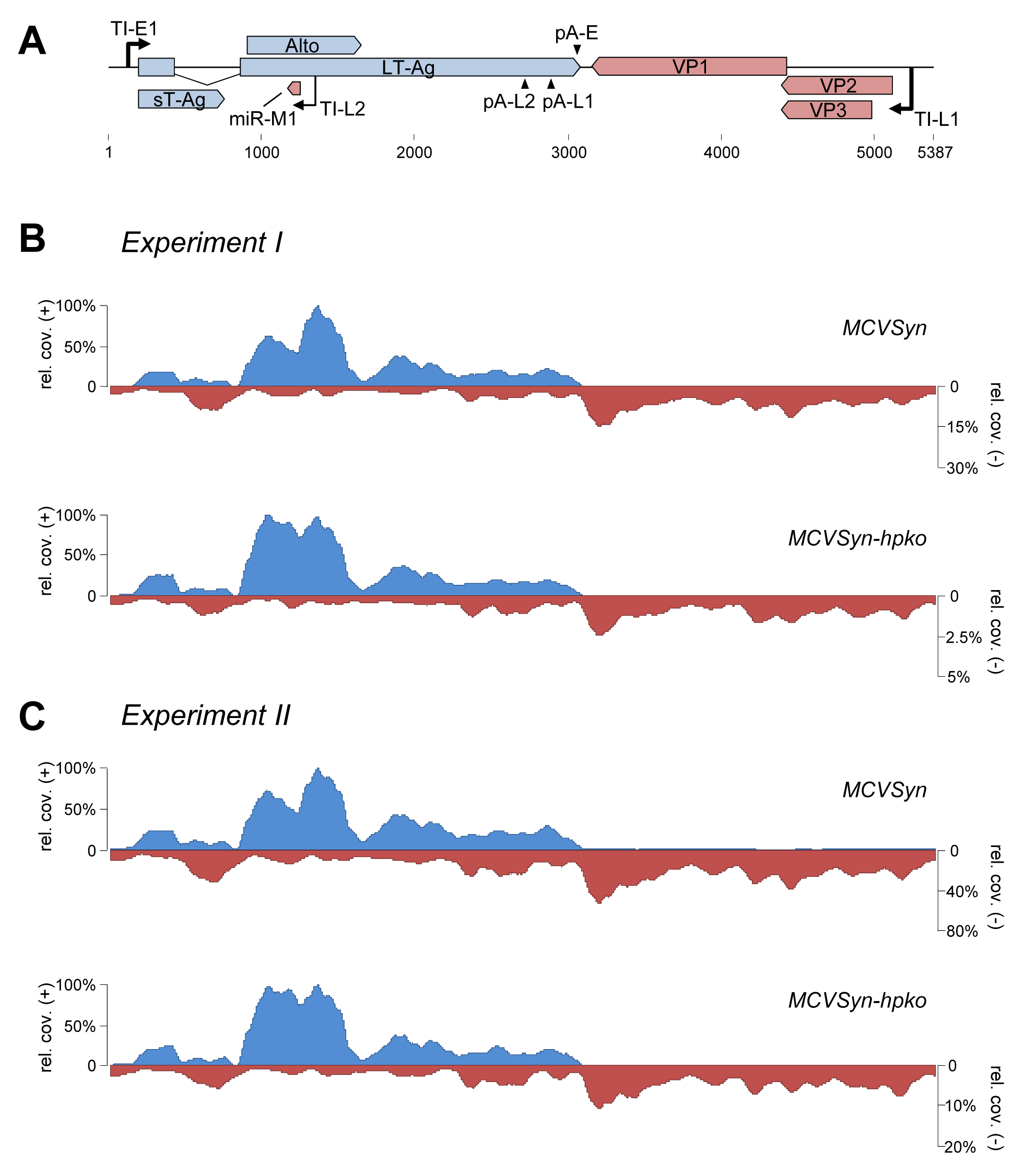

Supplement: S4 Fig — (A) Schematic depiction of the MCPyV genome. (B, C) RNA-seq coverage of PFSK-1 cells transfected for four days with either MCVSyn or MCVSyn-hpko (top and bottom graphs in each panel, respectively). A and B represent two independently performed experiments in which PFSK-1 cells were in parallel transfected with either wt or mutated genomes. Read coverage on the early (positive axis; blue) or late strand (negative axis; red) is shown relative to the maximally observed nucleotide coverage (set to 100%) across the viral genome. Note that the negative/late strand axis is shown at a lower scale for results from MCVSyn-hpko transfected cells to facilitate comparison of late strand coverage profiles. (TIF) [file ppat.1004974.s004.tif]

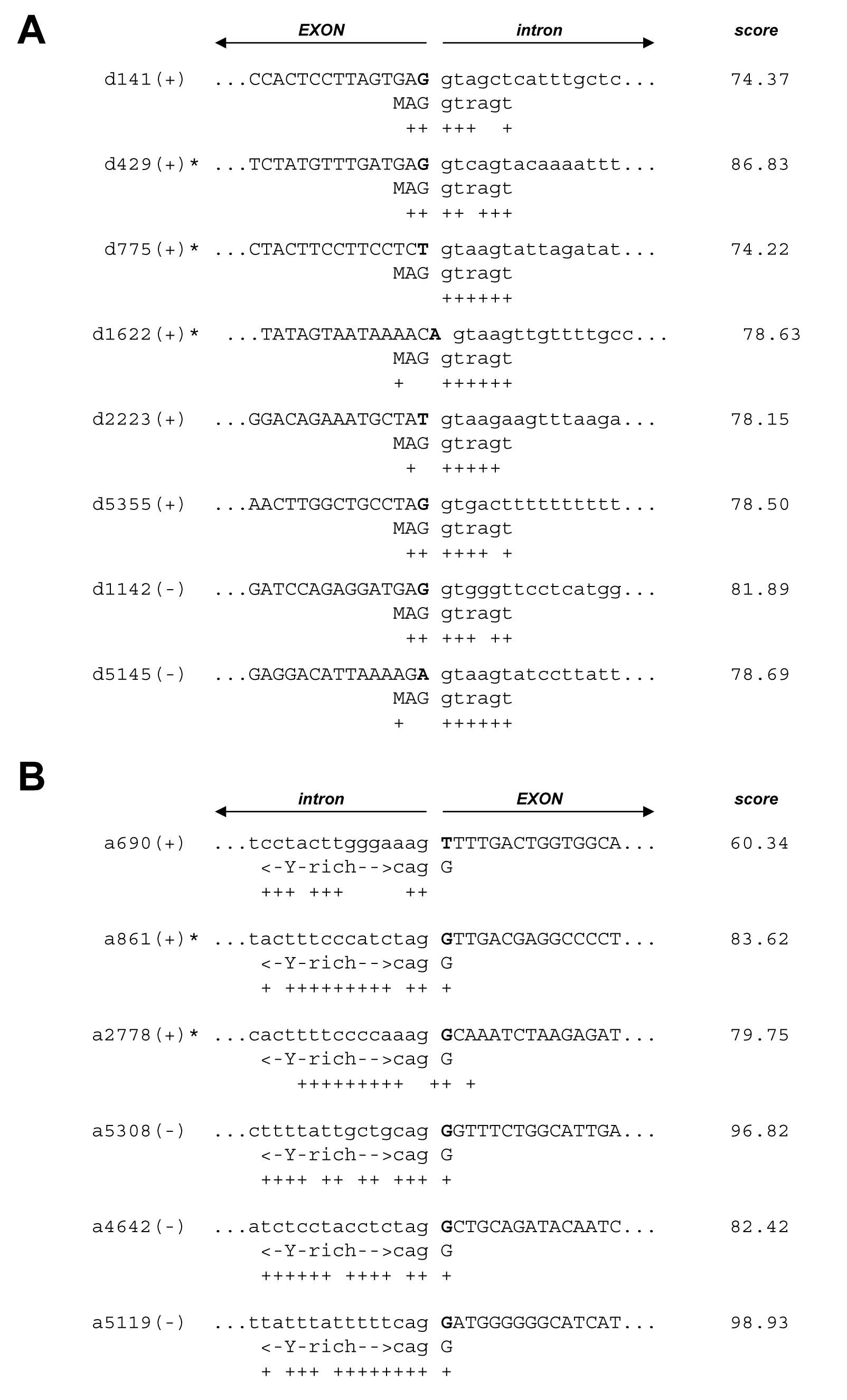

Supplement: S5 Fig — MCPyV donor (A) or acceptor (B) sites (shown in bold) identified by Shuda et al. (marked with an asterisk) [8] or in this study. Matches to consensus splice site sequences (shown underneath each donor or acceptor site) are indicated by plus signs. Scores shown to the right of each site were calculated using the AST tool (http://ibis.tau.ac.il/ssat/SpliceSiteFrame.htm). (TIF) [file ppat.1004974.s005.tif]

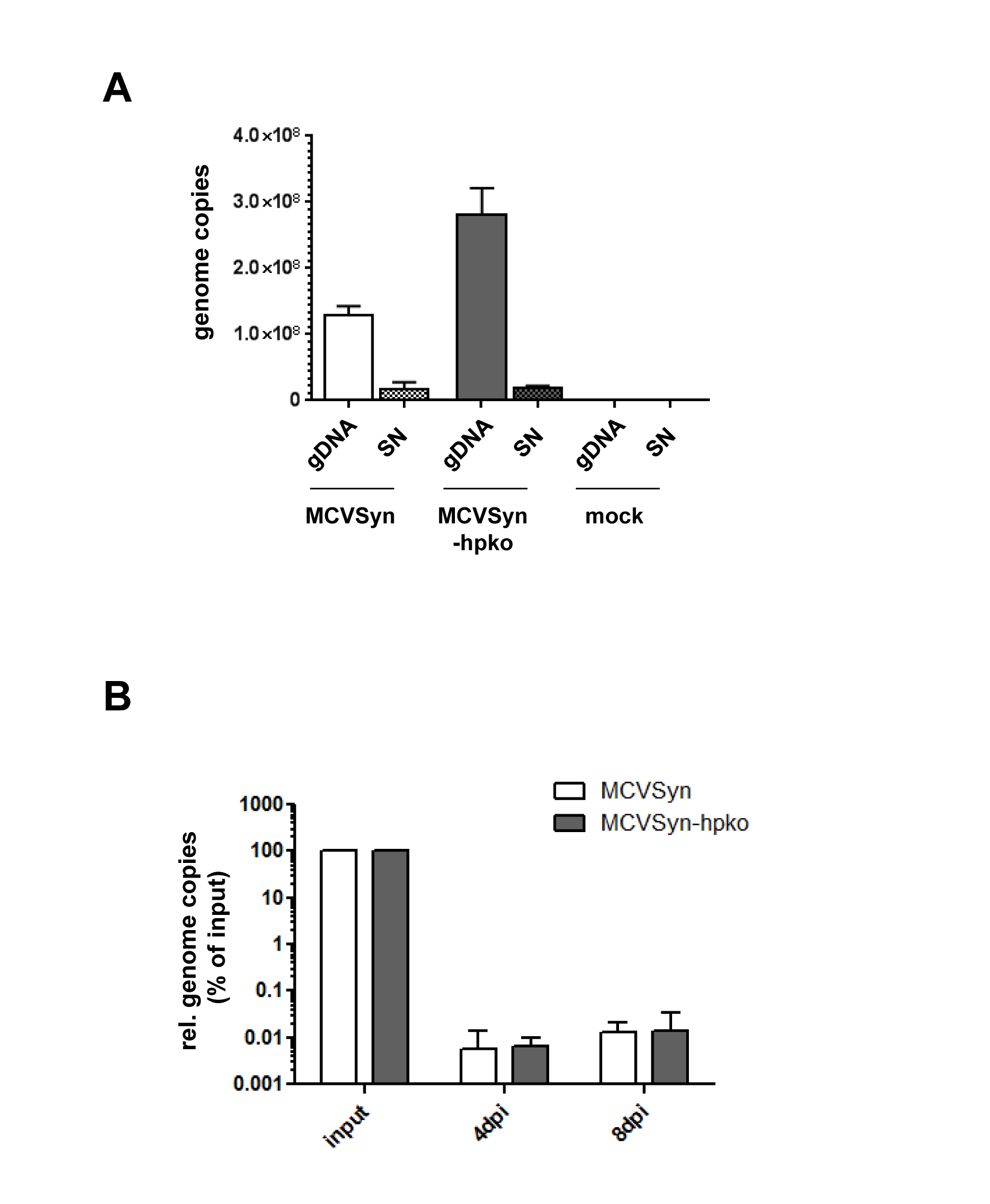

Supplement: S6 Fig — (A) Viral genome copy numbers from total genomic DNA (gDNA) or DNaseI treated supernatants (SN) of PFSK-1 cells at 4d after transfection with MCVSyn or MCVSyn-hpko. Mean values and standard deviations were calculated from three independent experiments. (B) Lysates prepared by freeze-thaw lysis from PFSK-1 cells at 8 days post transfection with MCVSyn wt or MCVSyn-hpko were used to inoculate fresh PFSK-1 cultures. MCVSyn copy numbers per cell were determined at 4d and 8d post infection by qPCR and are shown relative to copy numbers in the transfected cultures from which the lysates were derived (input, set to 100%). Mean values and standard deviations were calculated from 3 independently performed experiments. (TIF) [file ppat.1004974.s006.tif]

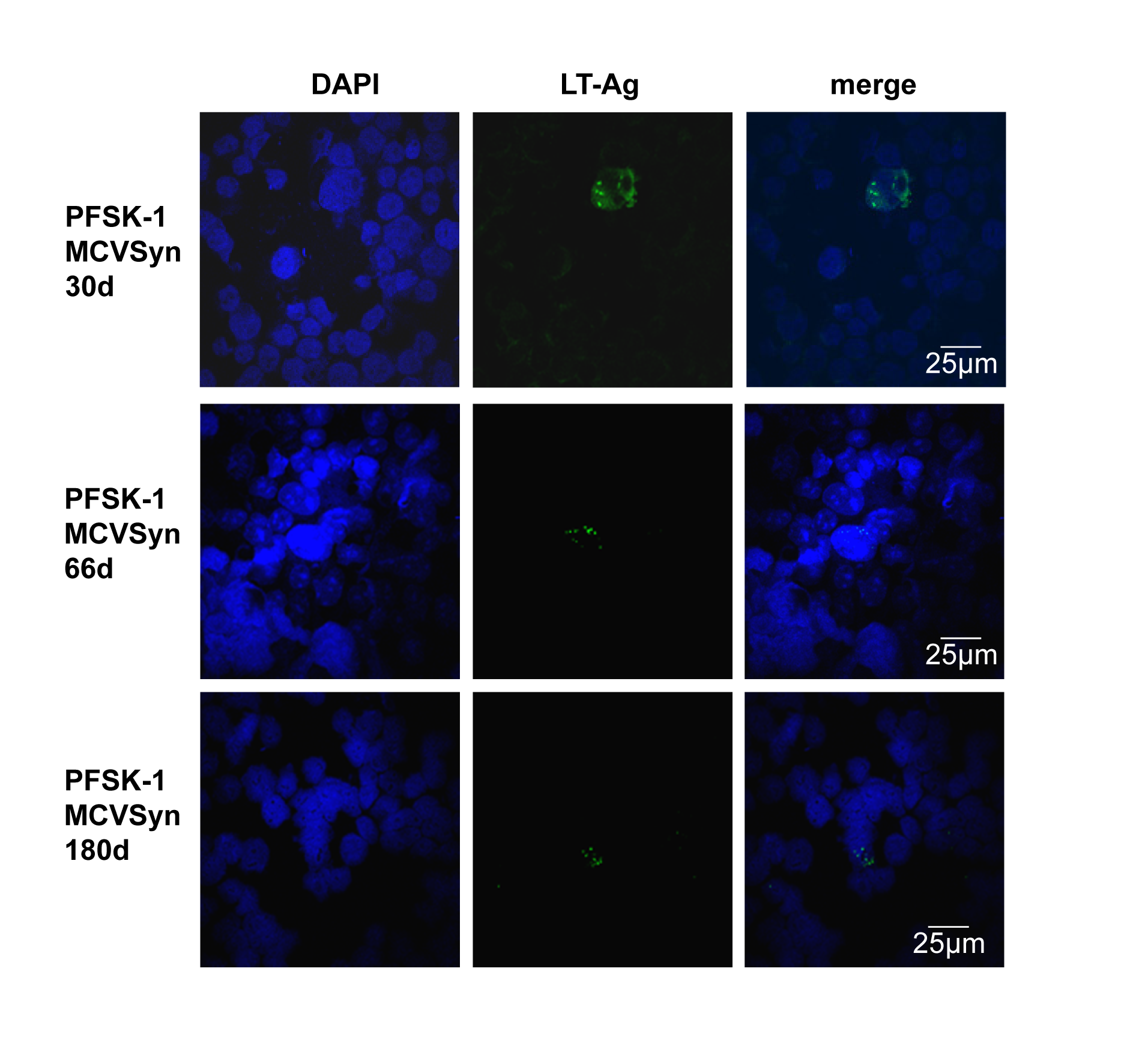

Supplement: S7 Fig — Shown are exemplary lower magnification FISH images derived from PFSK-1 cultures at the indicated time points after transfection with MCVSyn. (TIF) [file ppat.1004974.s007.tif]

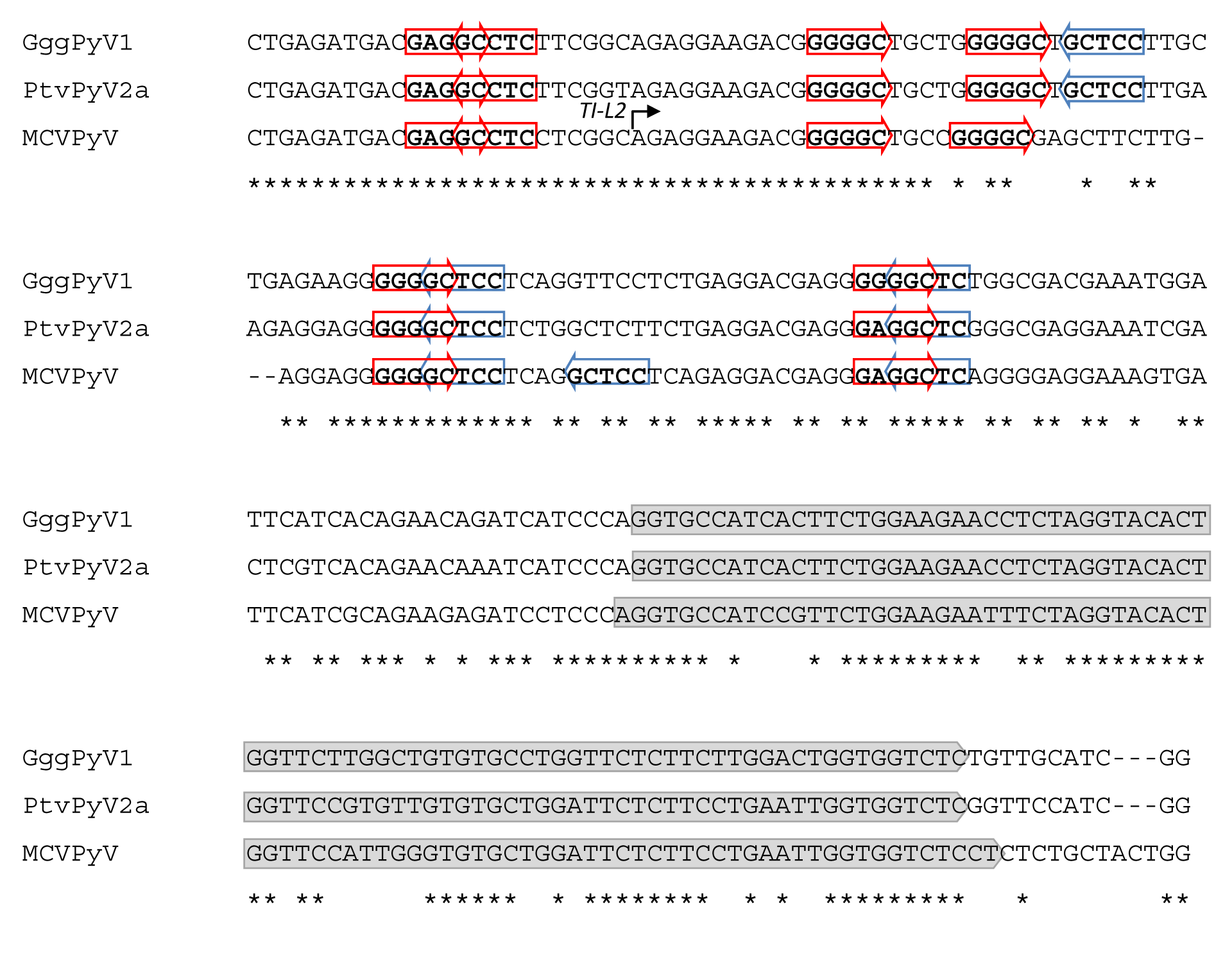

Supplement: S8 Fig — Alignments show genomic sequences upstream of the miRNA loci (shaded grey) in MCPyV and the related polyomaviruses Gorilla gorilla polyomavirus 1 (GggPyV1) and Pan troglodytes verus polyomavirus 2 (PtvPyV2a) [64, 65]. GRGGC pentamers are shown in bold and marked by red block arrows. Additional GGAGC or GAGCC sequences as observed at the imperfect P3 site of the MCPyV origin of replication [41, 42] are marked by blue block arrows. The location of the transcriptional initiation site TI-L2 is marked with a solid arrow. (TIF) [file ppat.1004974.s008.tif]

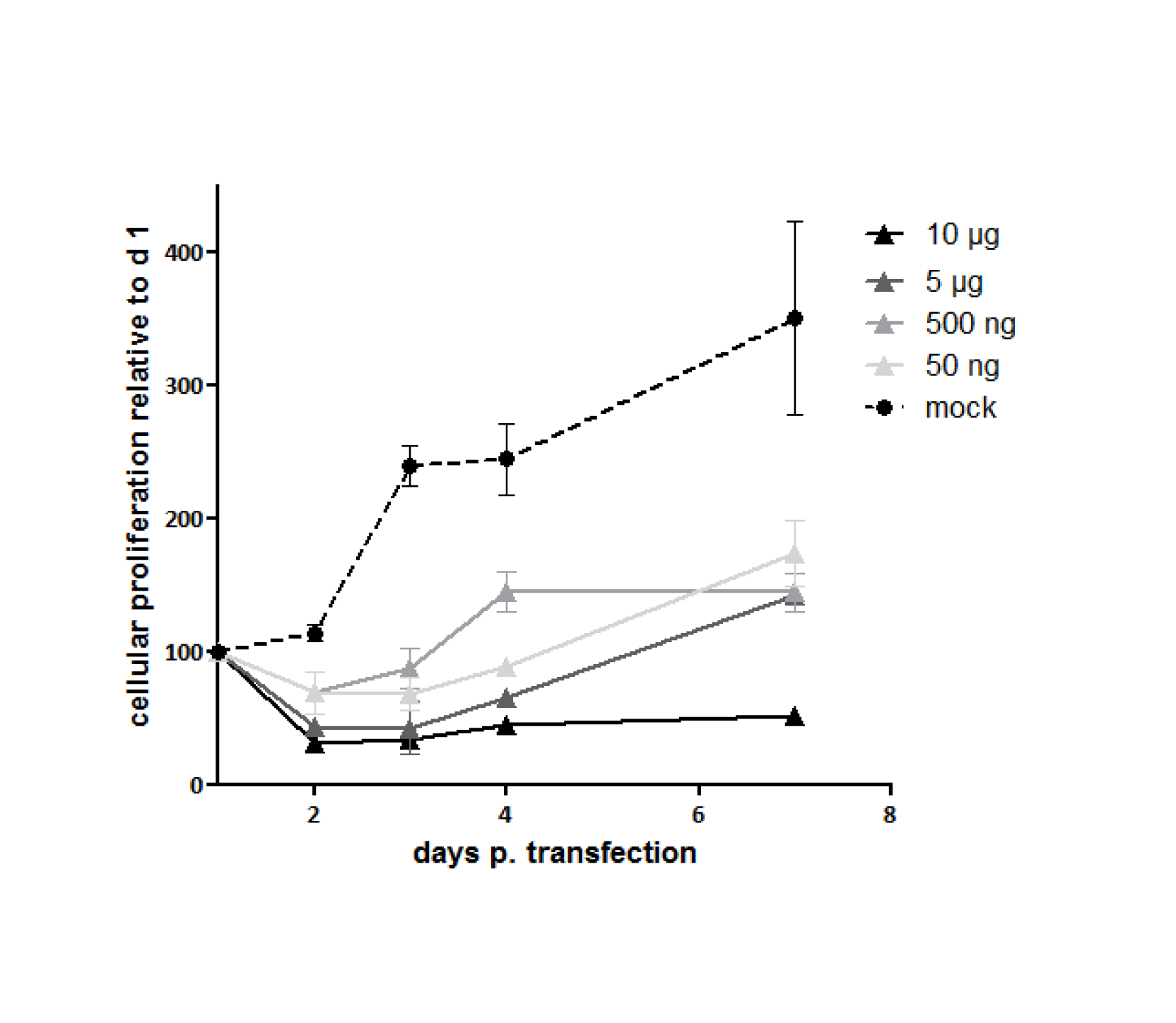

Supplement: S9 Fig — PFSK-1 cells were transfected with increasing amounts of a LT antigen expression construct (pCMV:ER-S) and proliferation was measured by MTT-assays at the indicated time points. Proliferation rates were calculated relative to the first measurement at 24h post transfection. The total amount of transfected DNA was brought to 10ug with pUC18 DNA in each transfection (‘mock’ hence indicates transfection of 10ug pUC 18 only). (TIF) [file ppat.1004974.s009.tif]
