# Supplementary material for: A Comprehensive Analysis of Replicating Merkel Cell Polyomavirus Genomes Delineates the Viral Transcription Program and Suggests a Role for mcv-miR-M1 in Episomal Persistence
Source: PLoS Pathog. 2015 Jul 28;11(7):e1004974. doi: 10.1371/journal.ppat.1004974 (PMC4517807; doi:10.1371/journal.ppat.1004974)
Supplement: S1 Table — (DOCX) [file ppat.1004974.s010.docx]

**S1 Table:** Primers and probes used in this study

| **primer/probe id** | **sequence (5'->3')** |
| --- | --- |
| MCPyV LT fw | CACACGGGACCAACTCAAG |
| MCPyV LT rev | AGGTATATCGGGTCCTCTG |
| MCPyV VP1 fw | AAAACACCCAAAAGGCAATG |
| MCPyV VP1 rev | GCAGAGACACTCTTGCCACA |
| GAPDH DNA fw | TGTGTCCCTCAATATGGTCCTGTC |
| GAPDH rev | ATGGTGGTGAAGACGCCAGT |
| mcv-miR-M1 probe | AGTGTACCTAGAAATTCTTCCAGAACGTATGGCAC |
| SL mcv-miR-M1 | GTCGTATCCAGTGCAGGGTCCGAGGTATTCGCACTGGATACGACTGTACC |
| mcv-miR-M1 fw | GCATCTGGAAGAATTTCTA |
| universal rev | GTGCAGGGTCCGAGGT |
| GAPDH BSP fw | GGTCGGAGTCAACGGATTTG |
| Taqman probe mcv-miR-M1 | (VIC^TM^)-CTGGATACGACAGTGTACC-(MGB) |
| Taqman probe GAPDH | (ROX^TM^)-GTGGCGCTGAGTACGTCGTGGAGTC-(MGB) |
| MCPyV EcoRV F | CCGATATCATGGATTTAGTCCTAAATAGG |
| MCPyV XhoI R | GGGCTCGAGTTGAGAAAAAGTACCAGAATCTTG |
| 3' RACE QT | CCAGTGAGCAGAGTGACGAGGACTCGAGCTCAAGCTTTTTTTTTTTTTTTTTVN |
| 3' RACE Qo | CCAGTGAGCAGAGTGACG |
| 3' RACE Qi | GAGGACTCGAGCTCAAGC |
| 3' RACE LTo | GGTGCTTGCCTGATACAACC |
| 3' RACE LTi | GTCTAGAGGATCCTTGCTTAC |
| 3' RACE VP1o | GGGGTCAGAACAATTACCTGG |
| 3' RACE VP1i | GTCTAGAGGATCCTGATATTG |
| 5' RACE RNA Adapter | GUUCAGAGUUCUACAGUCCGA |
| 5‘ RACE Adapter Primer | GTTCAGAGTTCTACAGTCCGA |
| 5‘ RACE Adapter Primer BamHI | AGGATCCGTTCAGAGTTCTACAGTC |
| 5‘ RACE late region rev | AGTGACACTTGCTCGCGTGACAACC |
| 5‘ RACE late rev nested | ACTCGAGCCACAGTTATTAGAGAGC |
| 5‘ RACE early region rev | ACGCTGAGAAGGACCCATACCCAGA |
| 5‘ RACE early rev nested | ACTCGAGAAGGCTTTCTGGATCTTGAG |
| 5‘ RACE miR-M1 3p hp rev | GGAAGCTCACCACCCCACAGCCAGA |
| 5‘ RACE miR-M1 3p nested XhoI | ACTCGAGATATACCTCCCGAACACCAT |
| 5‘ RACE miR-M1 5p hp rev | GGAGGCCTCGTCATCTCAGTTTACA |
| 5‘ RACE miR-M1 5p nested XhoI | ACTCGAGAAGAAAGCGAAAATTTGGGG |
| d5335-a861 BSP fw | TTAGTGAGGTTGACGAGGCC |
| d5335-a861 rev | AGGTATATCGGGTCCTCTG |
| d141-a861 BSP fw | CTTGGCTGCCTAGGTTGAC |
| d141-a861 rev | GACGCTGAGAAGGACCCATA |
| d1142-a5308 BSP fw | AGAGGATGAGGGTTTCTGGC |
| d1142-a5308 rev | TCCTGTGGTGGCACTTAGTT |
| d5145-a5308 fw | CTTTCTGTTTGGGAGGGAG |
| d5145-a5308 BSP rev | CCAGAAACCTCTTTTAATGTC |
